# Recent applications of ring-rearrangement metathesis in organic synthesis

**DOI:** 10.3762/bjoc.11.199

**Published:** 2015-10-07

**Authors:** Sambasivarao Kotha, Milind Meshram, Priti Khedkar, Shaibal Banerjee, Deepak Deodhar

**Affiliations:** 1 Department of Chemistry, Indian Institute of Technology Bombay, Powai, Mumbai-400 076, India. Fax: (+91)-22-2572-3480 2576 7152; Phone: (+91)-22-2576-7160; 2 Department of Chemistry, Guru Nanak Khalsa College of Arts, Science & Commerce, Matunga, Mumbai–400 019, India; 3 Department of Applied Chemistry, Defence Institute of Advanced Technology (DU), Girinagar, Pune–411025, Pune, India

**Keywords:** Diels–Alder chemistry, green chemistry, natural products, olefin metathesis, polycycles, ring-rearrangement metathesis

## Abstract

Ring-rearrangement metathesis (RRM) involves multiple metathesis processes such as ring-opening metathesis (ROM)/ring-closing metathesis (RCM) in a one-pot operation to generate complex targets. RRM delivers complex frameworks that are difficult to assemble by conventional methods. The noteworthy point about this type of protocol is multi-bond formation and it is an atom economic process. In this review, we have covered literature that appeared during the last seven years (2008–2014).

## Introduction

**Figure 1:**
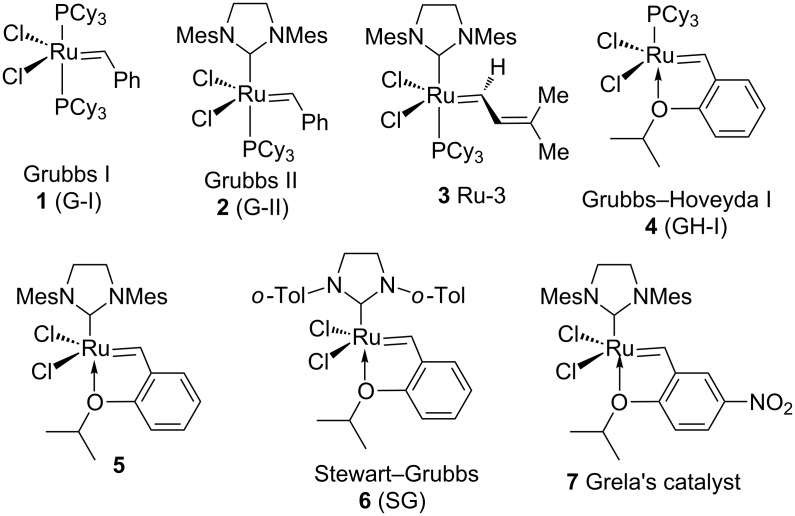
Ruthenium alkylidene catalysts used in RRM processes.

**Figure 2:**
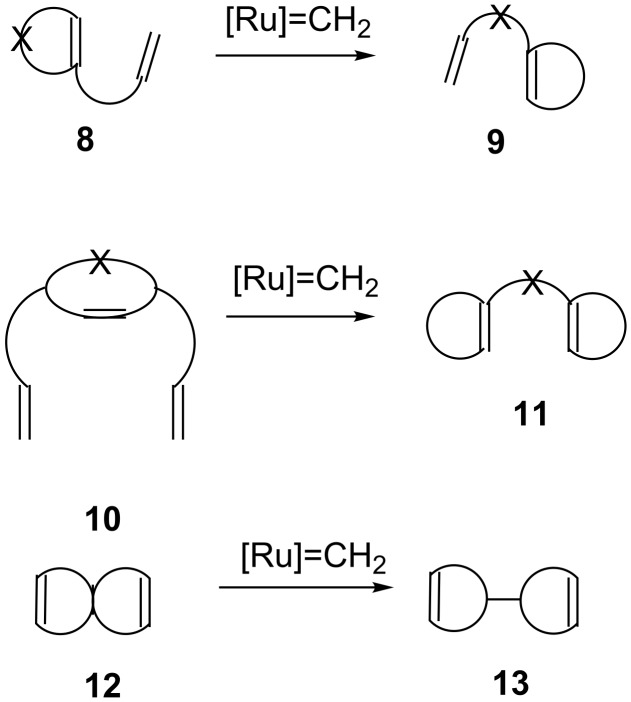
General representation of various RRM processes.

**Figure 3:**
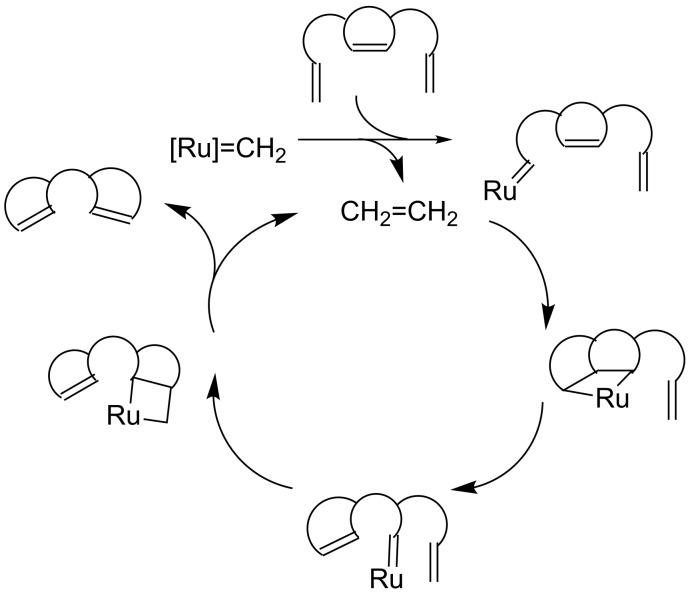
A general mechanism for RRM process.

Transition metal–carbene complexes (Figure 1) introduced during the last two decades have changed the landscape of organic synthesis. Armed with these advances, olefin metathesis has become a staple in retrosynthesis. Metathesis protocols such as ring-closing metathesis (RCM), cross-metathesis (CM), and enyne metathesis (EM) have gained popularity in the synthesis of complex molecules. Ring-rearrangement metathesis (RRM) involves a tandem process, where the ring-opening metathesis (ROM) and the RCM sequence occur in tandem to generate complex end products (Figure 2). Several demanding structures related to natural products and non-natural products were synthesized by RRM. However, a limited number of papers appeared dealing with RRM due to the complexity involved in designing the required precursors suitable for RRM. There are several factors which facilitates the RRM. Among them, the release of ring strain is the main driving force. For example, with bicyclo[2.2.1]heptene systems, RRM produce less strained end products. A general mechanism for the RRM process is shown in Figure 3 [1–2]. During RRM the stereochemical information is transformed from the substrate to the product. Interestingly, RRM is applicable to mono- and polycyclic systems of varying ring sizes. The outcome of the RRM process depends on the selection of the protecting groups, reaction conditions, and electronic properties of substrates involved. Oligomerization is a common side reaction in the RRM and external olefins such as ethylene prevents unwanted oligomerization processes. For earlier work related to the RRM readers may refer to excellent reviews available in the literature [3–6].

## Review

### Cyclopropene systems

**Scheme 1:**
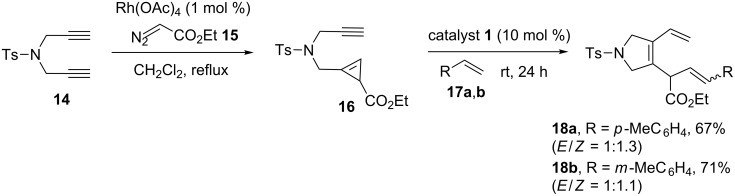
RRM of cyclopropene systems.

Cyclopropene derivatives are highly strained systems and they are ideal candidates for the RRM process. In this context, Zhu and Shi [7] have reported the ring-closing enyne metathesis (RCEM) of small-rings such as cyclopropenes by employing the Grubbs’ first-generation (G-I) catalyst. They have reported a new tandem ROM–RCM–CM sequence starting with 1,6-cyclopropenynes **16** with a wide variety of substituted olefins. To this end, the required building block **16** has been prepared with the aid of a carbene insertion reaction. Further, this cyclopropene system **16** was subjected to RRM in the presence of catalyst **1** to generate 3-pyrroline derivatives **18a,b** using simple starting materials in a single step (Scheme 1).

**Scheme 2:**
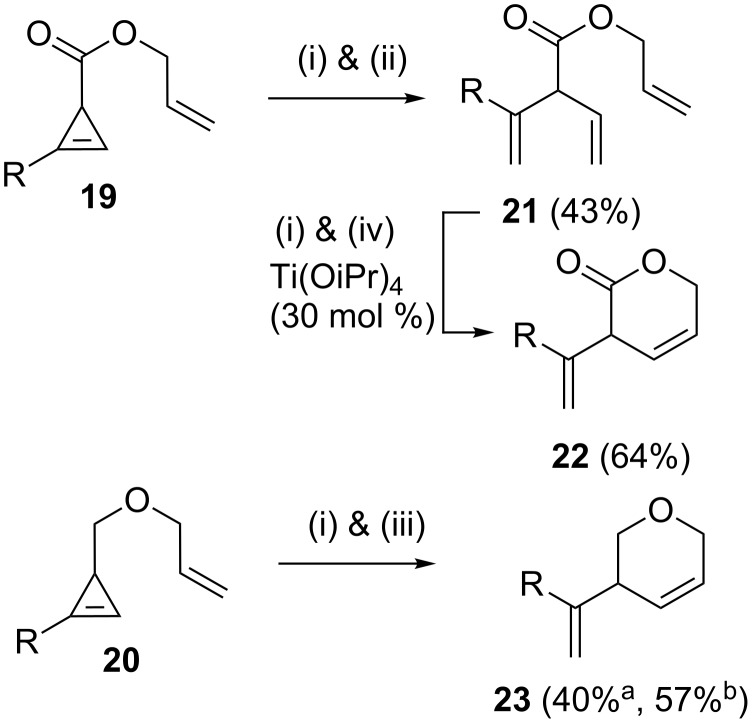
RRM of cyclopropene with catalyst **2**. (i) catalyst **2** (2.5 mol %), ethylene (**24**, 1 atm), (ii) toluene (*c* = 0.02 M), reflux, (iii) CH_2_Cl_2_ (*c* = 0.02 M), reflux, (iv) C_6_H_6_ (*c* = 0.01 M), reflux. (a) without ethylene (**24**); (b) with ethylene (**24**).

**Scheme 3:**
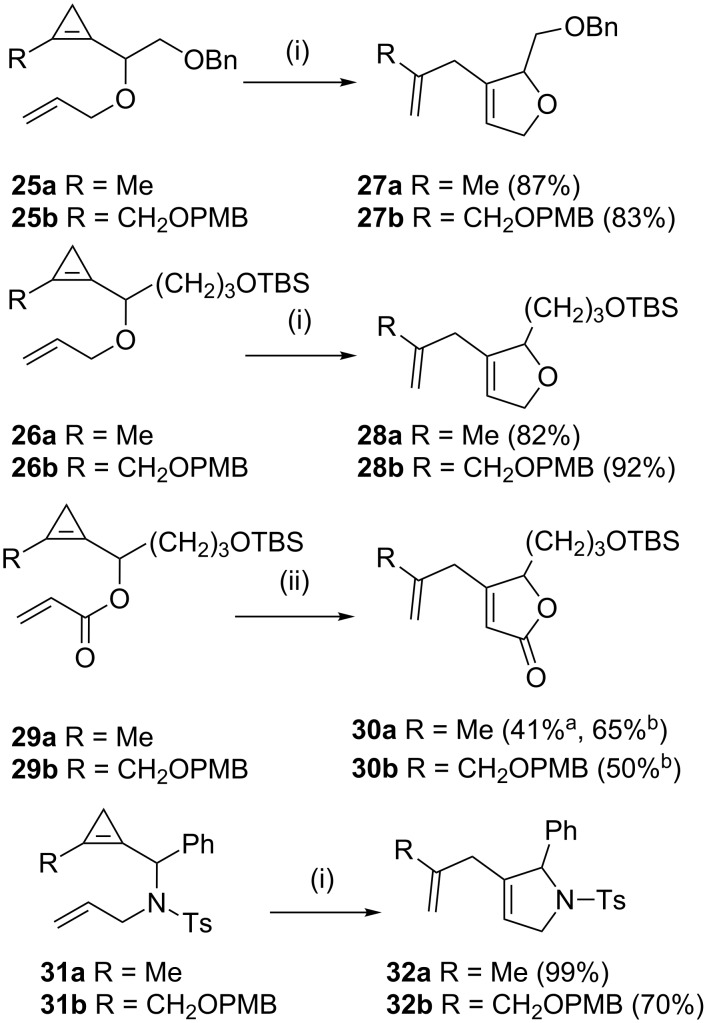
RRM of various cyclopropene derivatives with catalyst **2**. (i) catalyst **2** (2.5 mol %), CH_2_Cl_2_ (*c* = 0.1 M), reflux, (ii) (a) catalyst **2** (2.5 mol %), toluene (*c* = 0.1 M), reflux, (b) catalyst **7**, toluene (*c* = 0.1 M), reflux.

**Scheme 4:**
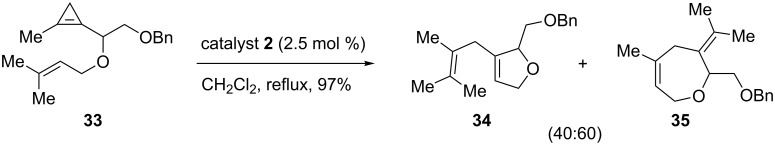
RRM of substituted cyclopropene system with catalyst **2**.

A wide range of heterocycles have been assembled by RRM. When a substituted cyclopropene such as **19** (or **20**) was treated with catalyst **2** in the presence of ethylene (**24**) the required heterocycle **22** (or **23**) was obtained in moderate to good yield (Scheme 2) [8]. Allyl ethers **25a**,**b** and **26a**,**b** were reacted with catalyst **2** to deliver the corresponding dihydrofurans (**27a**,**b** and **28a**,**b**) in excellent yields (82–92%). Involvement of acrylates **29a**,**b** delivered lactones **30a**,**b** in moderate yields (**30a** 41%, **30b** 50%) upon treatment with catalyst **2** in dichloromethane at reflux conditions. However, **29a** generated lactone **30a** in 65% yield when the metathesis was performed using Grela’s catalyst **7**. Pyrrolines were produced in excellent yields by RRM of sulfonamides **31a**,**b** using the catalyst **2** under dichloromethane reflux conditions (**32a** 99%, **32b** 70%) (Scheme 3). Five-membered heterocycles such as **34** and a seven membered heterocycle **35** in 40:60 ratio (97%) were formed by RRM of cyclopropenylcarbinyl ether **33** with catalyst **2** (Scheme 4).

### Cyclobutene systems

**Scheme 5:**
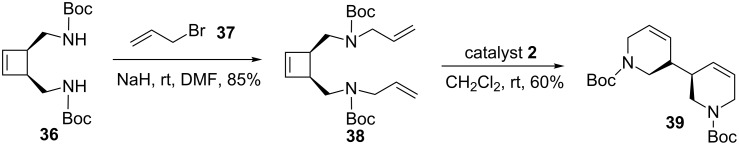
RRM of cyclobutene system with catalyst **2**.

Cyclobutene is also highly strained and prone to RRM very easily. Maougal and co-workers synthesized 3,3’-bipiperidine and 3,3’-bis(1,2,3,6-tetrahydropyridine) systems through a RRM sequence [9]. In this context, they have identified compound **38** as the key starting synthone, easily prepared from **36** via an *N*-allylation sequence. Next, diallyl compound **38** was treated with catalyst **2** to deliver the expected bipiperidine derivative **39** in 60% yield. Further, this protocol has been extended to various oxygenated systems (Scheme 5).

**Scheme 6:**
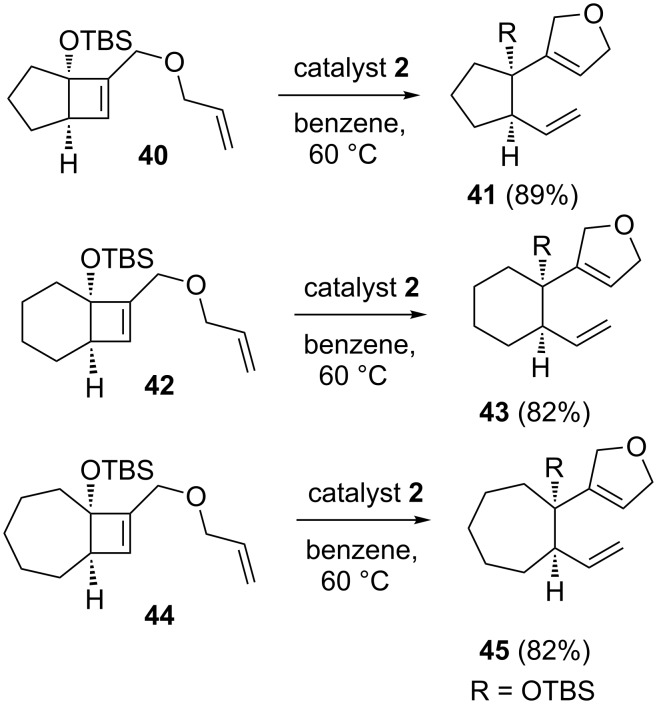
RRM approach to various bicyclic compounds.

Snapper and White [10] have reported a new and efficient method to various medium size bicyclic systems. Here, the RRM strategy has been employed with catalyst **2** starting with various cyclobutene systems containing an alkene tether (e.g., **40**, **42**, and **44**) to generate bicyclic systems such as **41**, **43**, and **45** (Scheme 6).

**Scheme 7:**
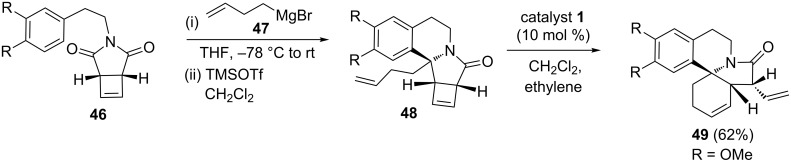
RRM approach to erythrina alkaloid framework.

The erythrina alkaloids are known to exhibit sedative, hypotensive and neuromuscular activity. This alkaloid skeleton consists of a tetracyclic spiroamine framework and synthetic chemists consider it as a challenging target. Simpkins and co-workers [11] have used the RRM sequence tactically to assemble the erythrina skeleton. To this end, they have identified cyclobutene derivative **48** as a useful synthone for RRM. The cyclobutene derivative **46** has been extended via Grignard addition followed by cyclization reaction. Later, cyclobutene derivative **48** was treated with catalyst **1** in the presence of ethylene (**24**) under high dilution conditions to deliver the tetracyclic compound **49** in 62% yield (Scheme 7).

**Scheme 8:**
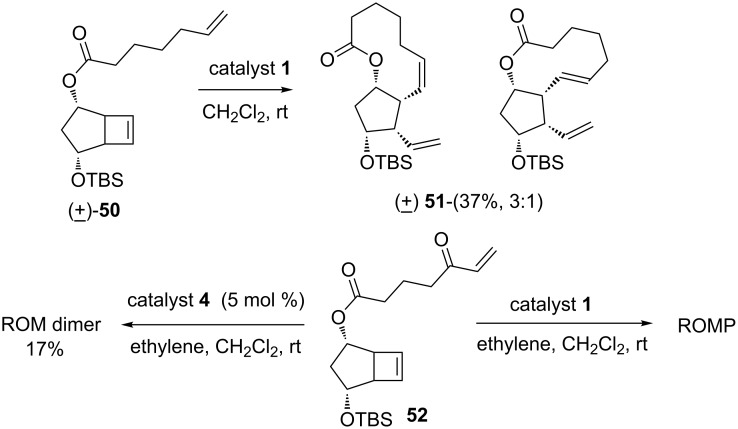
ROM–RCM sequence to lactone derivatives.

To assemble 5-F_2_-isoprostanes, lipid oxidation metabolites, various functionalized cyclobutene derivatives were subjected to a RRM sequence [12]. Cyclobutene derivative **50** in the presence of the catalyst **1** delivered lactone **51** as a mixture of isomers (3:1) in 37% yield. When the substrate was modified as in **52**, the RCM product was not formed; however, compound **52** gave the ring-opened product with ethylene (**24**) in low yield. Further, the ROM homodimer was obtained in 17% yield in the presence of ethylene (**24**) with the aid of catalyst **4** (Scheme 8).

**Scheme 9:**
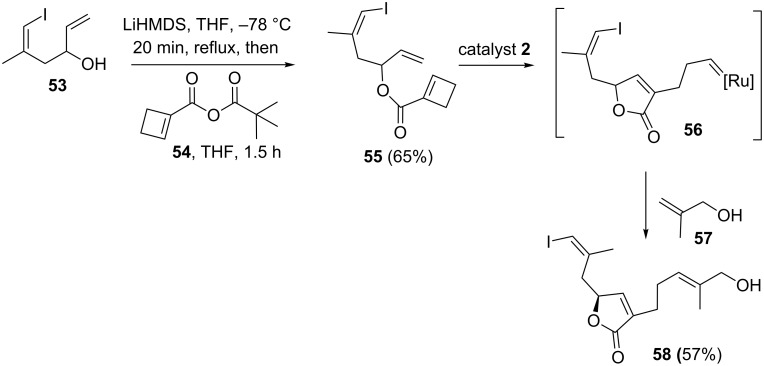
RRM protocol towards the synthesis of lactone derivative **58**.

Pattenden and co-workers [13] have described a novel synthesis of (+)-*Z*-deoxypukalide using substituted butenolide intermediate **58**. Interestingly, it was synthesized starting with cyclobutene ester **55** involving ROM–RCM and CM protocols. In this regard, the cyclobutene ester was subjected to a ROM–RCM and CM protocol under conditions with catalyst **2** in the presence of 2-methylpropenol **57** to afford the required butenolide intermediate **58** in 57% yield (Scheme 9).

**Scheme 10:**
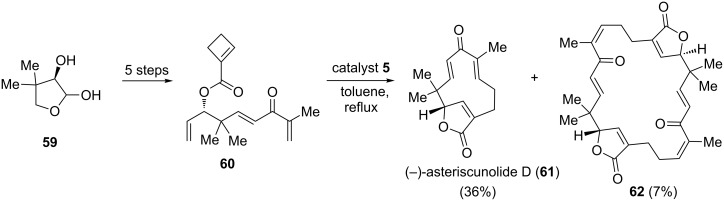
RRM protocol towards the asymmetric synthesis of asteriscunolide D (**61**).

An asymmetric synthesis of humulanolides is achieved by a RRM approach. In this context, Li and co-workers [14] prepared the key precursor **60** in five steps from commercially available starting material **59**. Later, the cyclobutene derivative **60** was treated with catalyst **5** under toluene reflux conditions to give the expected RRM cascade product, i.e., asteriscunolide D (**61**) in 36% yield along with the dimer **62** (7%). Interestingly, they also found asteriscunolide D as a useful synthone for the synthesis of asteriscunolides A−C (Scheme 10).

**Scheme 11:**
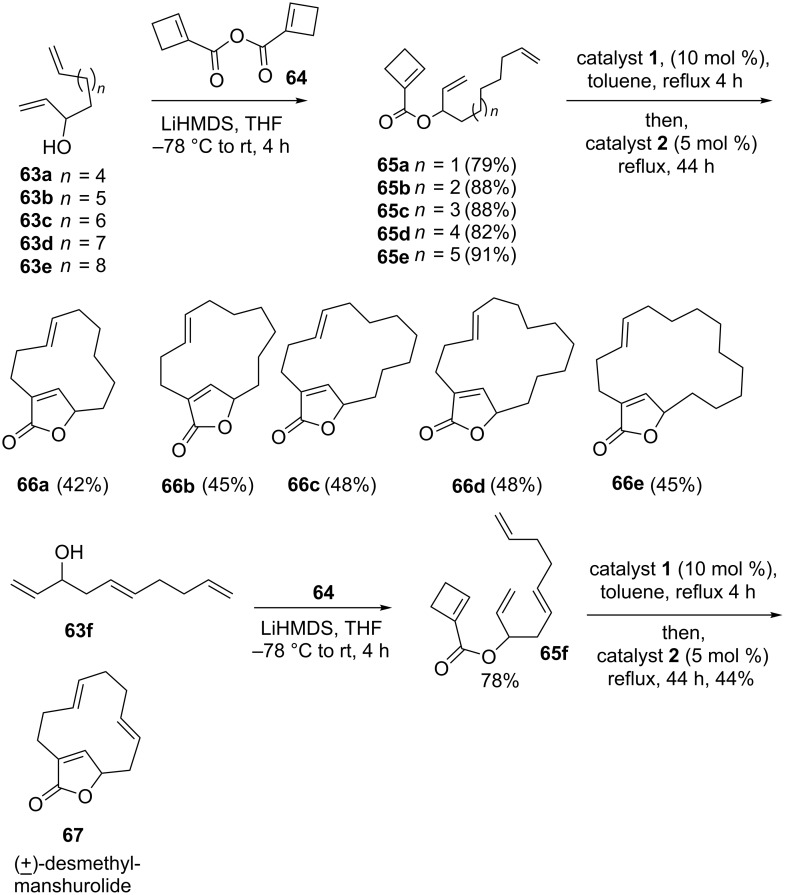
RRM strategy towards the synthesis of various macrolide rings.

In several instances RRM has proved to be a useful strategy for the construction of 12- to 16-membered macrolides [15]. In this regard, ester **65** was prepared from the corresponding allylic alcohol **63** by esterification with the anhydride **64** derived from cyclobutene. Later, the ester **65**, on treatment with the catalyst **1** under toluene reflux conditions followed by treatment with the catalyst **2** furnished the macrolide-butenolides **66** in 42–48% yields via RRM with *E*-selectivity at the macrocyclic double bond. Along similar lines, compound **65f** was treated with catalyst **1** in refluxing toluene followed by treatment with catalyst **2** to deliver desmethylmanshurolide **67** in 44% yield (Scheme 11).

### Cyclopentene systems

**Scheme 12:**
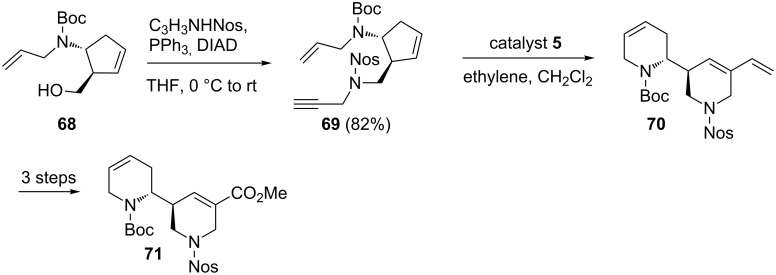
RRM protocol to dipiperidine system.

In RRM with cyclopentene systems, the release of ring strain is a less important contributor to the driving force of the reaction. However, unfavorable interaction of vicinal or proximal substituents may be minimized in the rearranged product. In this context, Blechert and co-workers [16] demonstrated the first enantioselective total syntheses of virgidivarine and virgiboidine by employing an intramolecular ene–ene–yne domino RRM protocol in combination with an oxidative C–C bond cleavage. This protocol opens-up new opportunities for the construction of intricate dipiperidine-based targets in a stereoselective manner (Scheme 12).

**Scheme 13:**
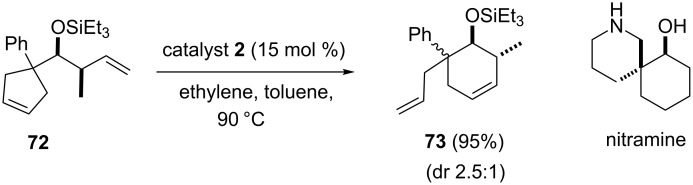
RRM of cyclopentene system to generate the cyclohexene systems.

Lee and Li [17] disclosed a highly distereoselective RRM approach starting with cyclopentene derivatives. In this regard, the cyclopentene derivative **72** was treated with the catalyst **2** in the presence of ethylene (**24**) to generate the required cyclohexene-based product **73**. The total synthesis of spiropiperidine alkaloid nitramine was proved to be efficient by this methodology (Scheme 13).

**Scheme 14:**
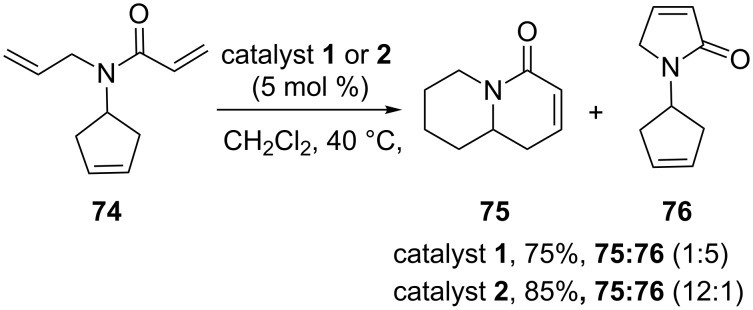
RRM of cyclopentene system **74**.

In 2004, Ni and Ma [18] have described the synthesis of bicyclic compounds **75** and **76** by adopting a metathesis protocol with catalysts **1** and **2** in good yields, but the product ratio is catalyst-dependent. In this context, when the cyclopentene derivative **74** gave **75** and **76** (1:5, 75%) with catalyst **1**; whereas, the catalyst **2** produced **75** and **76** in 85% yield (12:1). Here, they have shown the thermodynamically favored RRM leads to the formation of **75**, while kinetically favored RCM gave the product **76** (Scheme 14).

### Cyclohexene systems

**Scheme 15:**
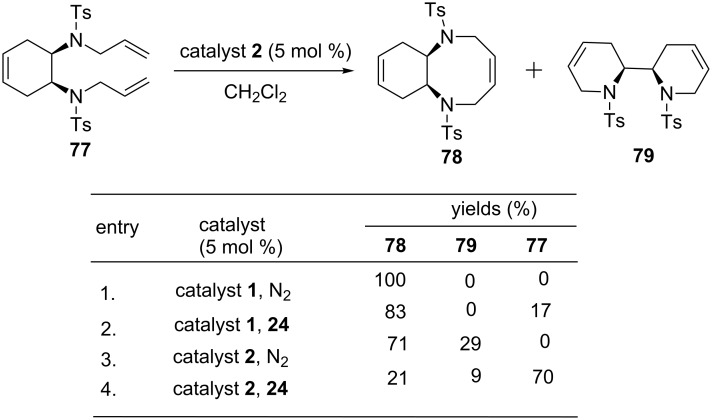
RRM approach to compound **79**.

Banti and co-workers have reported a tandem metathesis sequence with the aid of catalysts **1** and **2** starting with cyclohexene and norbornene systems containing allylamino moieties [19]. When the reaction was carried out in the presence of catalyst **2**; RRM product **79** was observed in 29% yield along with the RCM product **78** in 71% (Scheme 15).

**Scheme 16:**
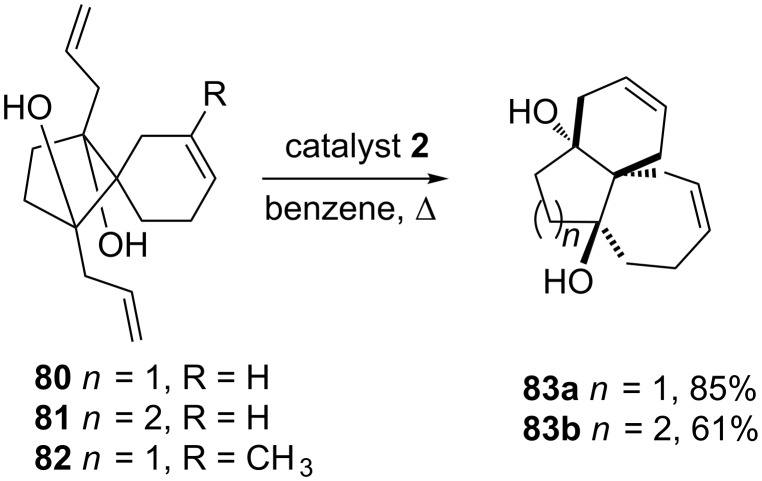
RRM approach to spirocycles.

Burnell and co-workers [20] have demonstrated the RRM of unsaturated spirocycles with two alkenyl chains by employing catalyst **2** to generate a unsaturated spiro-fused tricyclic system. In this context, the compounds **80** and **81** were subjected to RRM with catalyst **2** to furnish exclusively fused tricyclic systems **83a** and **83b** in 85% and 61% yields, respectively. Substitution on the cyclohexene system as in compound **82** did not deliver the RRM product (Scheme 16).

### Pyran systems

**Scheme 17:**
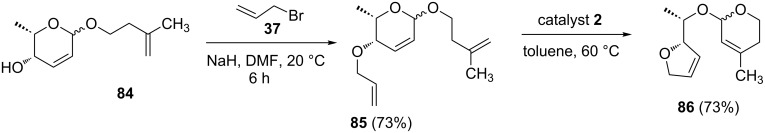
RRM approach to bicyclic dihydropyrans.

Donnard and co-workers [21] have accomplished a RRM approach for assembling complex heterocycles by employing simple starting materials. They have studied the RRM of dihydropyrans and dihydrofurans and this approach was found to be useful for the synthesis of non-classical saccharides. The synthesis of unusual di- or trisaccharides and related systems are also accessible by this approach. The required building block **85** has been prepared from compound **84** by allylation with allyl bromide (**37**). Later, the pyran derivative **85** was treated with catalyst **2** to generate the bicyclic system **86** (73%) (Scheme 17).

**Scheme 18:**
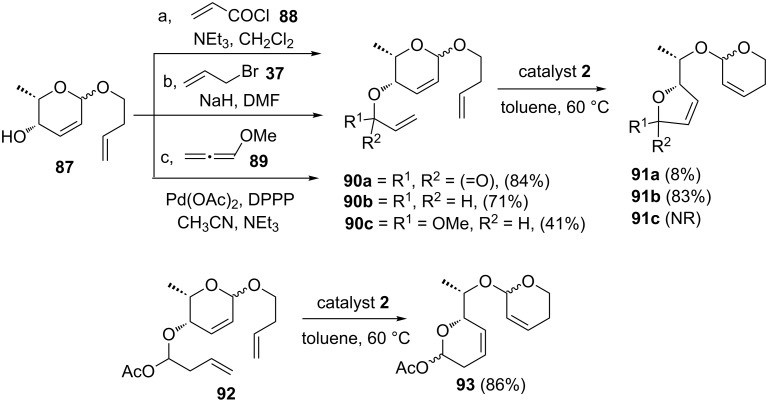
RCM–ROM–RCM cascade using non strained alkenyl heterocycles.

They also demonstrated a RCM–ROM–RCM cascade using a strain-free allyl heterocycle as useful starting material [22]. The required building blocks such as **90a–c** were prepared from compound **87**. Later, treatment of **90b** with catalyst **2** gave the expected RRM product **91b** (83%), whereas compound **90a** gave the rearranged product **91a** in 8% yield. On the other hand, when compound **90c** was reacted with catalyst **2** the rearranged product was not formed. Here, they have demonstrated that the success of the reaction depends on electronic and stereochemical factors. Moreover, the synthesis of unusual polydeoxydisaccharides could be achieved starting with these simple starting materials. Similarly, **93** has been obtained by the RRM of compound **92** (Scheme 18).

**Scheme 19:**
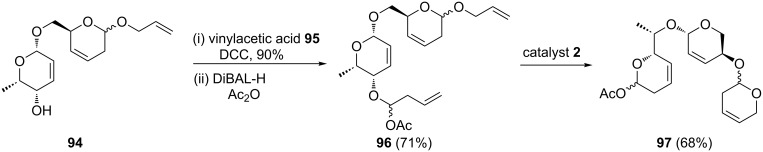
First ROM–RCM–ROM–RCM cascade for the synthesis of trisaccharide **97**.

Eustache and co-workers [23] have reported a novel ROM–RCM–ROM–RCM cascade involving a simple heterocycle as a useful precursor for the RRM protocol. To this end, the required precursor **96** was synthesized from **94** in two steps. Next, **96** was treated with catalyst **2** to generate the expected RRM product **97** in 68% yield. Further, this approach is useful for the preparation of polyunsaturated trisaccharides (Scheme 19).

**Scheme 20:**
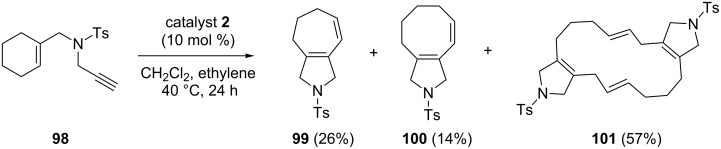
RRM of cyclohexene system.

Mori and co-workers [24] have used the RRM protocol starting with enyne **98** using catalyst **2** in the presence of ethylene (**24**) to generate the dimerized 16-membered ring product **101** in 57% yield, which was generated by a RRM–dimerization sequence and its monomer **100** in 14% yield along with **99** in 26% yield (Scheme 20).

### Bicyclo[2.2.1]heptene derivatives

**Scheme 21:**
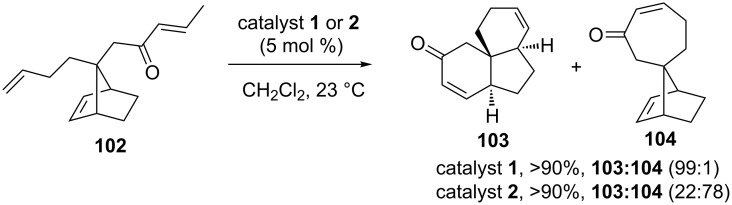
RRM approach to tricyclic spirosystem.

Holtsclaw and Koreeda [25] have explored a chemoselective RRM of the enone containing norbornene system such as **102**. To this end, the spironorbornene **102** was subjected to a RRM sequence under the influence of catalyst **1** to deliver the RRM product **103** and the RCM product **104** in a 99:1 ratio. When norbornene derivative **102** was treated with catalyst **2** tricyclic compound **103** and spironorbornene derivative **104** were obtained in a 22:78 ratio (Scheme 21).

**Scheme 22:**
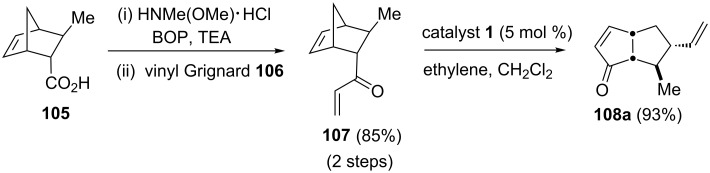
RRM approach to bicyclic building block **108a**.

Aubé and co-workers [26] have accomplished the asymmetric synthesis of the dendrobatid alkaloid 251F by employing a RRM as the key step. The required building block **108a** has been synthesized from enone **107** via a RRM protocol. When enone **107** was exposed to catalyst **1** in the presence of ethylene (**24**) the RRM product **108a** was obtained in 93% yield. Further, this bicyclic building block **108a** has been successfully utilized in the synthesis of the dendrobatid alkaloid 251F (Scheme 22).

**Scheme 23:**
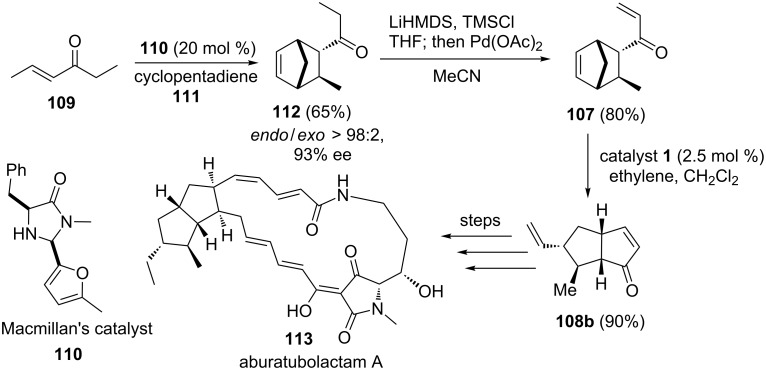
ROM–RCM protocol for the synthesis of the bicyclo[3.3.0]octene system.

Phillips and Henderson [27] have demonstrated the synthesis of aburatubolactam A (**113**) by using a tandem ROM–RCM sequence as the key step. To this end, the required key building block, the bicyclo[3.3.0]octene ring system **108b** has been synthesized by a RRM sequence via catalyst **1** starting with the functionalized bicyclo[2.2.1]heptene system **107**. Thus, the Diels–Alder (DA) reaction of ketone **109** with cyclopentadiene (**111**) in the presence of MacMillans catalyst **110** gave bicyclic ketone **112** in 65% yield. Then, ketone **112** was transformed into enone **107** in 80% yield by adopting the known oxidation protocol. Later, enone **107** was treated with catalyst **1** under ethylene (**24**) atmosphere to deliver the required bicyclo[3.3.0]octane derivative **108b** in 90% yield (Scheme 23).

**Scheme 24:**
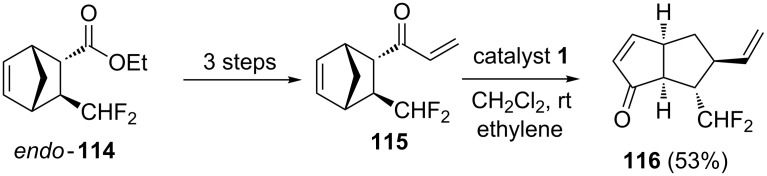
RRM protocol to bicyclic enone.

Shibatomi and co-workers have reported an enantioselective DA reaction of β-fluoromethylacrylate under the influence of the chiral Lewis acid-activated catalyst, oxazaborolidine to generate the difluoromethylated cycloaddition *endo*-product **114** (99% ee). Further, it was used to prepare the required enone **115**. Later, enone **115** was subjected to a RRM protocol by employing catalyst **1** in the presence of ethylene (**24**) to generate the bicyclic enone **116** in 53% yield (Scheme 24) [28].

**Scheme 25:**
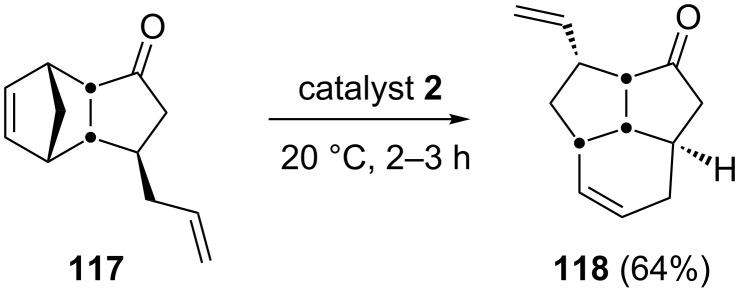
RRM protocol toward the synthesis of the tricyclic system **118**.

In 2005, Funel and Prunet have disclosed the synthesis of fused tricyclic systems by employing a RRM protocol [29]. For example, the bicyclic system **117** was treated with catalyst **2** to generate the rearranged tricyclic system **118**. This strategy has been extended with higher analogues (Scheme 25).

**Scheme 26:**
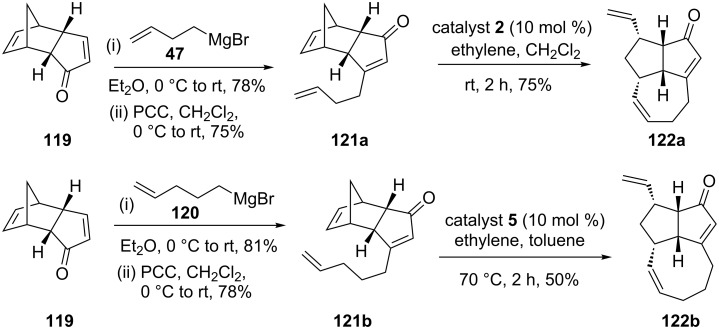
RRM approach toward the synthesis of the tricyclic enones **122a** and **122b**.

Kotha and Ravikumar [30] have utilized the RRM and the enyne RRM to generate various polycyclic scaffolds. In this context, enones, such as **121a** and **121b** were assembled easily from dicyclopentadiene derivative **119**. Later, these componds were subjected to a RRM to generate the tricyclic enones **122a** and **122b**, respectively. To this end, compound **121a** was treated with catalyst **2** under ethylene (**24**) atmosphere to deliver the tricyclic enone **122a** in 75% yield. Similarly, the tricyclic compound **122b** (50%) was obtained under the influence of catalyst **5** in the presence of ethylene (**24**, Scheme 26).

**Scheme 27:**
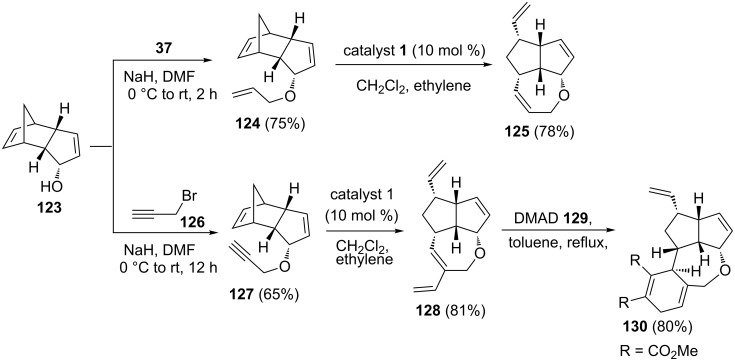
Synthesis of tricyclic and tetracyclic systems via RRM protocol.

Along similar lines, the oxa analog **125** was obtained by RRM of **124** using catalyst **1** under ethylene (**24**) atmosphere. Interestingly, the diene building block **128**, produced by employing an enyne-ring rearrangement metathesis (ERRM) sequence, was subjected to a DA reaction in refluxing toluene with various dienophiles such as dimethyl acetylenedicarboxylate (DMAD, **129)** to generate the tetracyclic system **130** (Scheme 27).

**Scheme 28:**
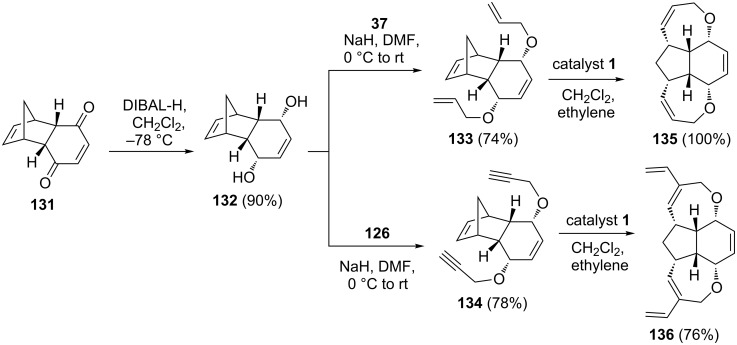
RRM protocol towards the synthesis of tetracyclic systems.

To design synthetically challenging oxa-bowls, Kotha and Ravikumar [31] have utilized the RRM and ERRM of extended norbornene systems. The key building blocks such as **133** and **134** were prepared from a readily available DA adduct **131** derived from cyclopentadiene (**111**) and 1,4-benzoquinone. The diol **132** was produced by reduction of **131** in an efficient manner. To this end, the bis-*O*-allylated compound **133** was prepared by an allylation sequence using allyl bromide (**37**) in the presence of NaH starting with the diol **132**, whereas compound **134** was derived via bis-*O*-propargylation of compound **132** using propargyl bromide (**126**) under similar reaction conditions. Later, these compounds (**133** and **134**) were subjected to RRM and ERRM protocols under the influence of catalyst **1** in the presence of ethylene (**24**) to generate the tetracyclic systems **135** (100%) and **136** (76%), respectively. Moreover, this strategy can easily be extended to other complex systems (Scheme 28).

**Scheme 29:**
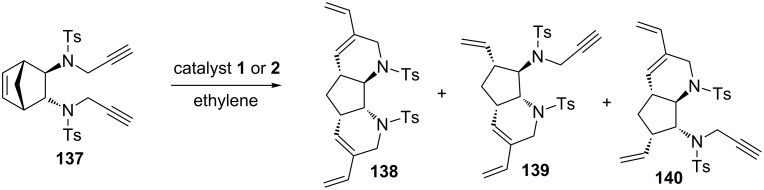
RRM of the propargylamino[2.2.1] system.

Banti and co-workers have described a RRM with catalysts **1** and **2** by using an aminopropargylated norbornene system as a starting material [19]. In this reaction, three possible products were observed by employing either catalyst **1** or **2**. The norbornene derivative **137** was subjected to a RRM protocol under the influence of catalyst **1** in the presence of ethylene (**24**) to obtain the expected product **138** in 41% yield (Table 1, entry 1) along with the *cis*- and *trans*-monocyclized products **139** and **140**. Further, NMR spectroscopic studies showed the presence of products **139** and **140** as a mixture of isomers, and it was difficult to purify this mixture by conventional separation techniques (Scheme 29).

**Table 1:** RRM of propargylamino derivative.

Entry	Cat (mol %)	Solvent	*T* (°C)	time (h)	Conv.	**138** (yield %)

1	**1** (5)	CH_2_Cl_2_	25	6	100	41
2	**1** (5)	CH_2_Cl_2_	25	16	100	43
3	**1** (5) + **2** (5)	CH_2_Cl_2_	25	24	100	37
4	**2** (5)	toluene	60	24	0	0

**Scheme 30:**
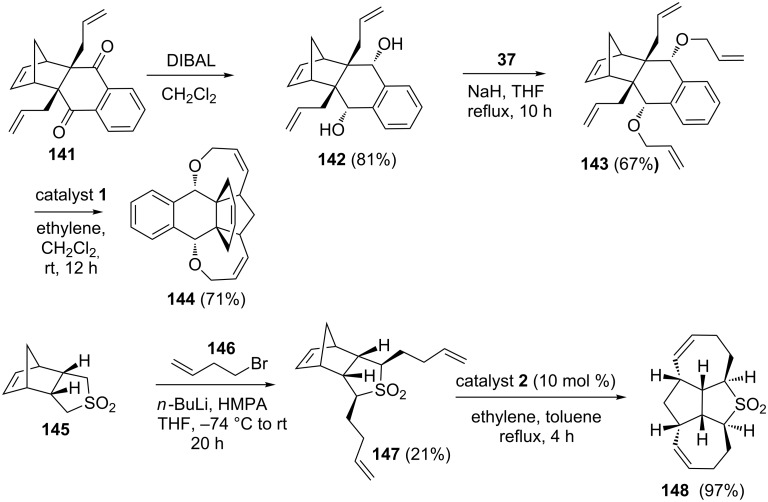
RRM of highly decorated bicyclo[2.2.1] systems.

Recently, Kotha and Gunta have reported a RRM to generate various polycyclic compounds using catalysts **1** and **2** [32]. The tetraallyl derivative, prepared from **142** by an allylation protocol, was subjected to a RRM sequence in the presence of the catalyst **1** to produce propellane derivative **144** containing an oxa-bowl moiety. In another sequence [33], the alkenylation of sulfone **145** gave the dialkenylated product **147** in 21% yield along with the monoalkenylated product. Later, the dialkenylated compound **147** was treated with catalyst **2** to give the tetracyclic compound **148** in 97% yield (Scheme 30).

**Scheme 31:**
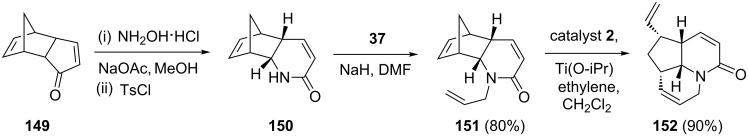
RRM protocol towards fused tricyclic compounds.

Along similar lines, Kotha and co-workers [34] prepared *N*-allylated compounds and subjected them to a RRM to produce the tricyclic aza compound **152** in an excellent yield. The required synthone **151** was prepared by employing a Beckman rearrangement followed by a *N*-allylation sequence. Later, it was reacted with catalyst **2** in the presence of ethylene (**24**) to deliver the expected tricyclic product **152** in 90% yield (Scheme 31).

**Scheme 32:**
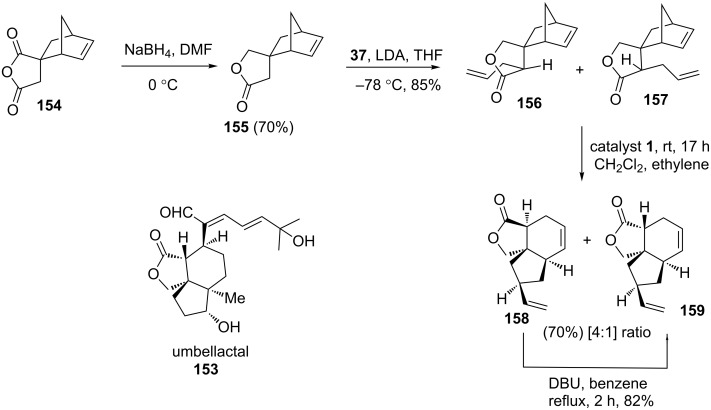
RRM protocol to functionalized tricyclic systems.

Ghosh and Maity [35–36] reported a stereoselective route to functionalized tricyclic system present in umbellactal (**153**) via a RRM protocol starting with intricate norbornene derivatives. The tricyclic anhydride **154** was reduced to lactone **155** using sodium borohydride and then it was monoallylated to deliver an inseparable mixture of products **156** and **157** in 85% combined yield. Later, the mixture (**156** and **157**) was subjected to a RRM protocol under the influence of the catalyst **1** in the presence of ethylene (**24**) to yield a mixture (4:1) of tricyclic lactones **158** and **159** (70% yield). Next, the major product **158** was converted into **159** by isomerization via DBU in 82% yield. The *cis*-lactone **159** was found to be a core structural unit present in umbellactal (Scheme 32).

**Scheme 33:**
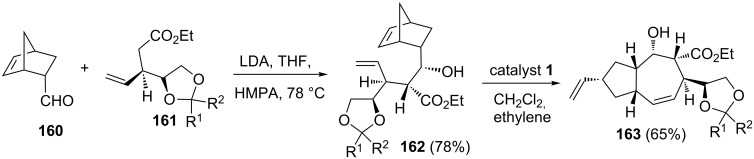
RRM approach to functionalized polycyclic systems.

Ghosh and co-workers also reported [37] a short and efficient approach to a highly functionalized lactarane skeleton using RRM with appropriate norbornene systems. The strategy starts with the aldol condensation of aldehyde **160** with ester **161** in the presence of LDA to generate the required building block **162** in 78% yield. Later, the norbornene derivative **162** was subjected to a RRM sequence under the influence of the catalyst **1** in the presence of ethylene (**24**) to produce the rearranged product **163** in 65% yield (Scheme 33).

**Scheme 34:**
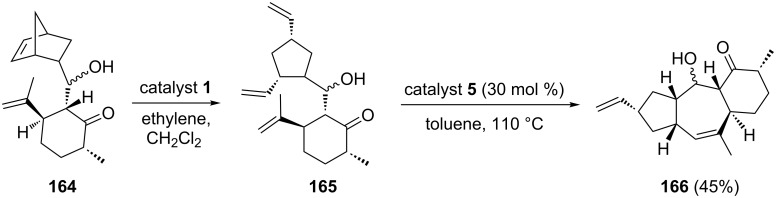
Sequential RRM approach to functionalized tricyclic ring system **166**.

Ghosh and co-workers have described an efficient route for the synthesis of the fused tricyclic system found in caribenol A by employing a RRM approach [38]. The steps employed here involve: a sequential aldol condensation of dihydrocarvone with norbornene 2-carboxaldehyde followed by a ROM–RCM of the resulting aldol product. The norbornene derivative **164** was subjected to a RRM using the catalyst **1** to produce the ROM product **165** exclusively. The ring-closure of the resulting ROM product **165** under the influence of the catalyst **2** led to the formation of the dimeric product. Alternatively, RCM of **165** under the influence of catalyst **5** generated the required tricyclic compound **166** in 45% yield (epimeric mixture at C-5). Interestingly, this tricyclic system was found as a core structural unit present in caribenol A (Scheme 34).

**Scheme 35:**
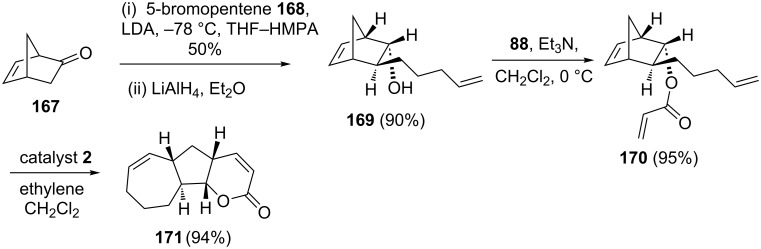
RRM protocol to functionalized CDE tricyclic ring system of schintrilactones A and B.

In another instance, they [39] achieved an efficient synthesis of the functionalized tricyclic ring system **171** in the context of the synthesis of the nonterpenoids schintrilactones A and B by a RRM approach of alkenylated norbornene derivative **170**. They also reported an impressive set of example with complex norbornene systems. The required synthone **170**, suitable for RRM, has been prepared from **167** in three steps. Later, compound **170** was treated with catalyst **2** in the presence of ethylene (**24**) to generate the desired tricyclic ring system **171** (94%), which is found to be a core structure of schintrilactones A and B (Scheme 35).

**Scheme 36:**
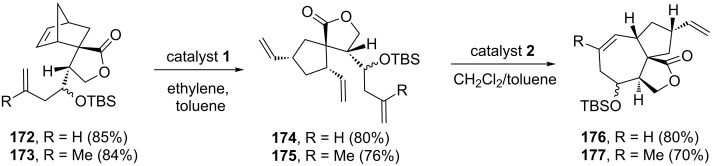
Sequential RRM approach to 7/5 fused bicyclic systems.

In 2012, Ghosh’s group [40] demonstrated a RRM approach towards the synthesis of a 7/5 fused system by using a bicyclo[2.2.1]heptene derivative via a sequential RRM approach. Moreover, they have studied the feasibility of a RRM protocol starting with highly substituted bicyclo[2.2.1]heptene and bicyclo[2.2.2]octene systems. Here, the silyl ether **172** was treated with catalyst **1** to give the ring-opened product **174**. Next, the triene **174** was subjected to a RCM protocol in the presence of catalyst **2** to furnish the tricyclic product **176**. Along similar lines, methyl substituted norbornene derivative **173** was treated with catalyst **1** in the presence of ethylene (**24**) to generate the ROM product **175**, which was further subjected to a RCM using catalyst **2** to deliver the expected tricyclic system **177** (7/5 fused system) (Scheme 36).

**Scheme 37:**
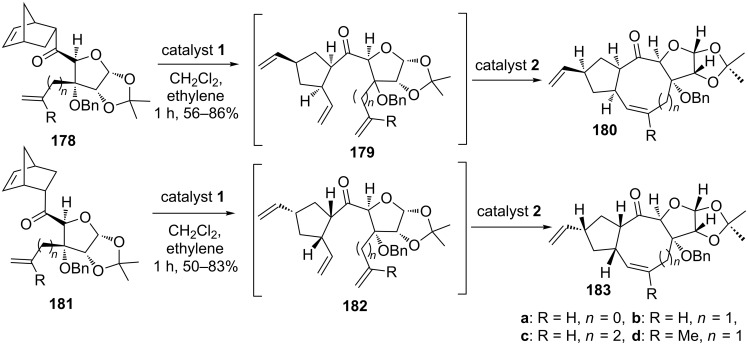
Sequential ROM-RCM protocol for the synthesis of bicyclic sugar derivatives.

A synthesis of fused medium-sized rings has been reported by Ghosh and co-workers [41] via a sequential diastereoselective DA reaction and a RRM protocol. A variety of sugar-based norbornene derivatives provide an entry to various functionalized bicyclic sugar derivatives containing 7–9 membered rings. To this end, compounds **178** and **181** were subjected to a ROM sequence with catalyst **1** in the presence of ethylene (**24**) followed by treatment with catalyst **2** under the same reaction conditions to give the RRM products **180** and **183**, respectively, derived from the ROM products. Here, the norbornene derivatives **178a**,**b**,**d** and **181a**,**c**,**d** furnished the RRM products **180a**,**b**,**d** and **183a**,**c**,**d**, respectively. As expected, when the compounds **178c** and **181b** were subjected to metathesis under the influence of the catalyst **1**, the RRM products (**180c** and **183b**) were obtained respectively (Scheme 37).

**Scheme 38:**
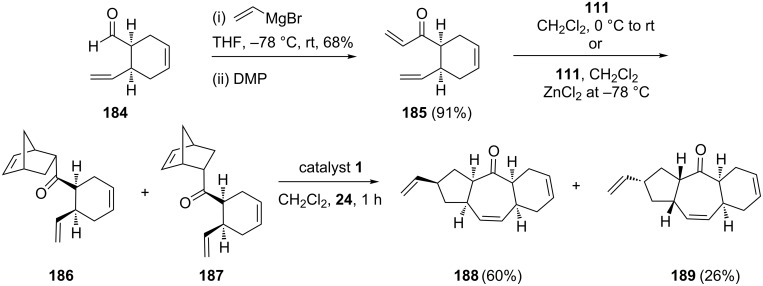
ROM–RCM sequence of the norbornene derivatives **186** and **187**.

Along similar lines, compound **185** was reacted with cyclopentadiene in a DA fashion to deliver an inseparable mixture of adducts **186** and **187**. Later, the ROM–RCM of this mixture of norbornene derivatives, gave the *cis-syn-cis* and *cis-anti-cis* 5-7-6 tricyclic systems **188** (60%) and **189** (26%), respectively, via the RRM approach (Scheme 38).

**Scheme 39:**
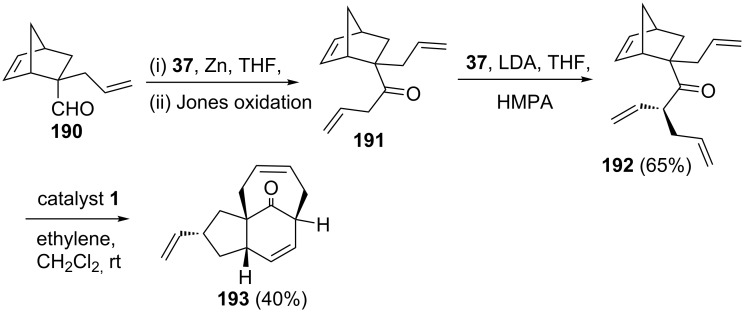
RRM approach toward highly functionalized bridge tricyclic system.

Ghosh’s group [42] disclosed an elegant approach to a highly functionalized bridged tricyclic system by employing a RRM approach involving catalyst **1**. The required synthone **192** has been prepared from the allyl substituted norbornene derivative **190** in three-steps. Later, the keto derivative **192** was subjected to a RRM sequence via catalyst **1** to generate the bridged tricyclic system **193** in 40% yield (Scheme 39).

**Scheme 40:**
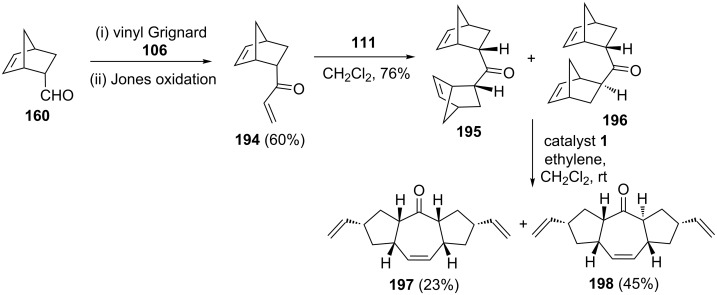
RRM approach toward highly functionalized tricyclic systems.

A novel approach to highly functionalized tricyclic systems such as **197** and **198** has been reported via a RRM protocol. In this context, the *endo*-aldehyde **160** was identified as a starting material in the synthetic sequence and it was transformed into enone **194** by treatment of vinyl Grignard **106** followed by Jones oxidation. Later, enone **194** was subjected to a DA reaction in the presence of cyclopentadiene (**111**) to deliver an inseparable mixture of cycloadducts **195** (*endo,endo*) and **196** (*exo,exo*) in a 1:2 ratio. Then, treatment of the cycloadducts **195** and **196** separately with catalyst **1** in the presence of ethylene (**24**) furnished the tricyclic compounds **197** (23%) and **198** (45%), respectively (Scheme 40). Analogously, they have also achieved the synthesis of angularly annelated carbocycles by employing the RRM protocol starting with appropriate norbornene derivatives [43].

**Scheme 41:**
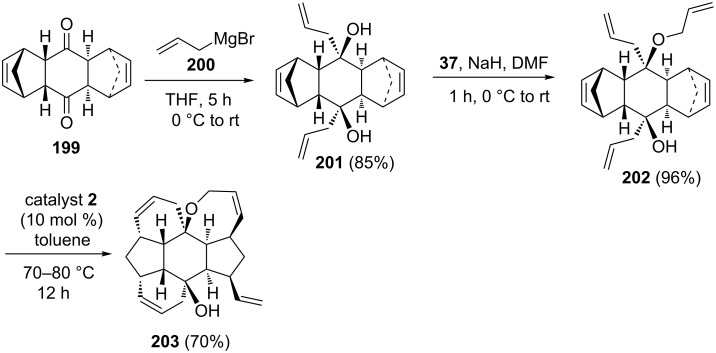
Synthesis of hexacyclic compound **203** by RRM approach.

Recently, Kotha and Ravikumar [44] have found a new route to various polycyclic compounds by employing the DA reaction and the RRM protocol as key steps. To this end, the key building block **202** has been prepared from **199** via Grignard addition followed by *O*-allylation. The double DA adduct **199** has been derived from cyclopentadiene and 1,4-benzoquinone. Next, compound **202** was exposed to catalyst **2** in the presence of ethylene (**24**) to generate the expected hexacyclic system **203** (70%) containing 10 stereogenic centres (Scheme 41).

**Scheme 42:**
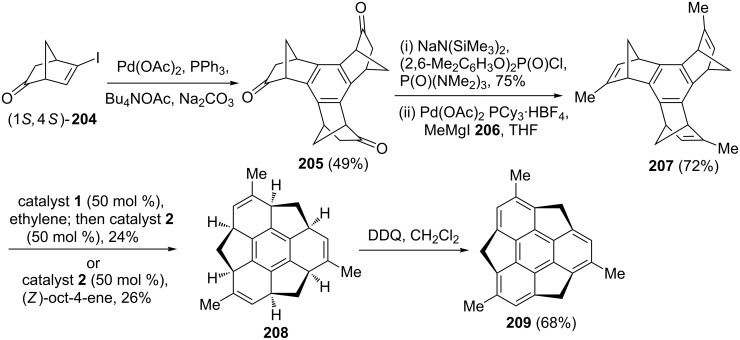
RRM approach toward *C*_3_-symmetric chiral trimethylsumanene **209**.

Sakurai and co-workers have successfully established an enantioselective synthesis of the *C*_3_-symmetric chiral trimethylsumanene through a Pd-catalyzed cyclotrimerization and the RRM protocol as key steps [45]. Here compound **207** reacted with catalyst **1** in the presence of ethylene (**24**) to deliver a mixture of ring-opened products. A sequential treatment with catalyst **2** resulted in a ring-closing product to deliver the expected hexahydrotrimethylsumanene **208** in 24% yield. When the tris-norbornene derivative **207** was treated with catalyst **2** in the presence of (*Z*)-oct-4-ene the required RRM product **208** was formed in 26% yield. Later, the expected chiral buckybowl **209** was assembled via aromatization of **208** in the presence of DDQ (Scheme 42).

**Scheme 43:**
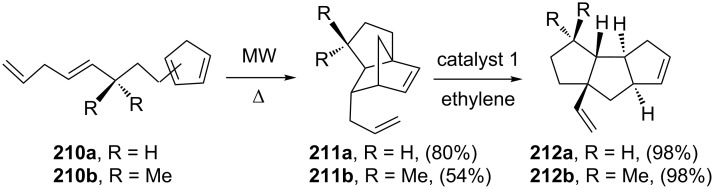
Triquinane synthesis via IMDA reaction and RRM protocol.

Design of intricate polyquinanes has been considered as a challenging task for synthetic chemists. To this end, Fallis and co-workers [46] have demonstrated an intramolecular Diels–Alder (IMDA) reaction followed by a RCM–ROM–CM cascade was found to be useful to assemble a linear triquinane framework. Microwave assisted IMDA reaction of cyclopentadiene derivative **210** performed in chlorobenzene at 201 °C under 310 psi pressure gave the required DA adduct **211**. Later, the cycloadduct **211** was reacted with the catalyst **1** in the presence ethylene (**24**) to generate a linear *cis-anti-cis* triquinane derivative **212** (Scheme 43).

**Scheme 44:**
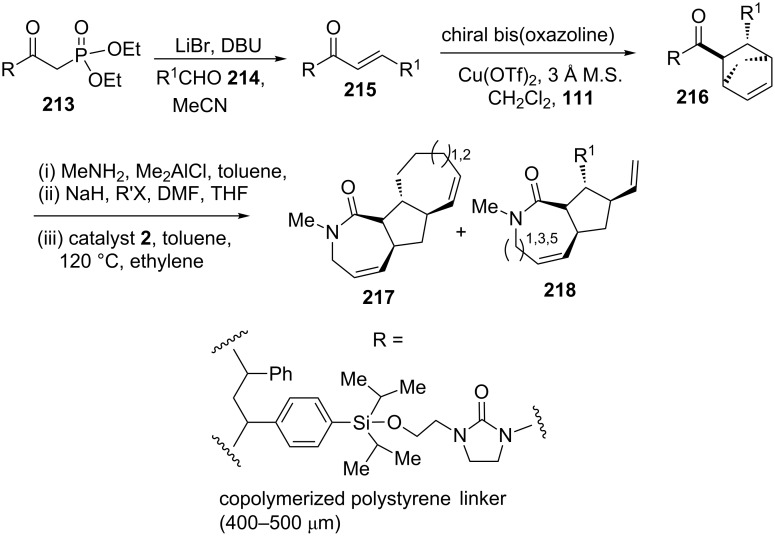
RRM approach to polycyclic compounds.

In search of new antibacterial drugs, Spring and co-workers [47] have designed a diversity-oriented approach to structurally diverse small molecules starting with solid-supported phosphonate **213**. In this regard, they have shown the use of a RRM protocol to prepare the bicyclic product **218** as well as tricyclic product **217**. To this end, the phosphonate ester **213** reacted with a wide variety of aldehydes **214** such as aryl, heteroaryl, and alkyl, etc. to produce α,β-unsaturated acylimidazolidinones **215**. Next, the Evan’s asymmetric DA methodology involving a [4 + 2] cycloaddition of chiral bis(oxazoline) in the presence of Cu(OTf)_2_ was employed to furnish the required norbornene system **216**. Later, it was converted into a lactam and then subjected to a RRM sequence with catalyst **2** in the presence of ethylene (**24**) to furnish the tricyclic product **217** as well as bicyclic product **218** (Scheme 44).

**Scheme 45:**
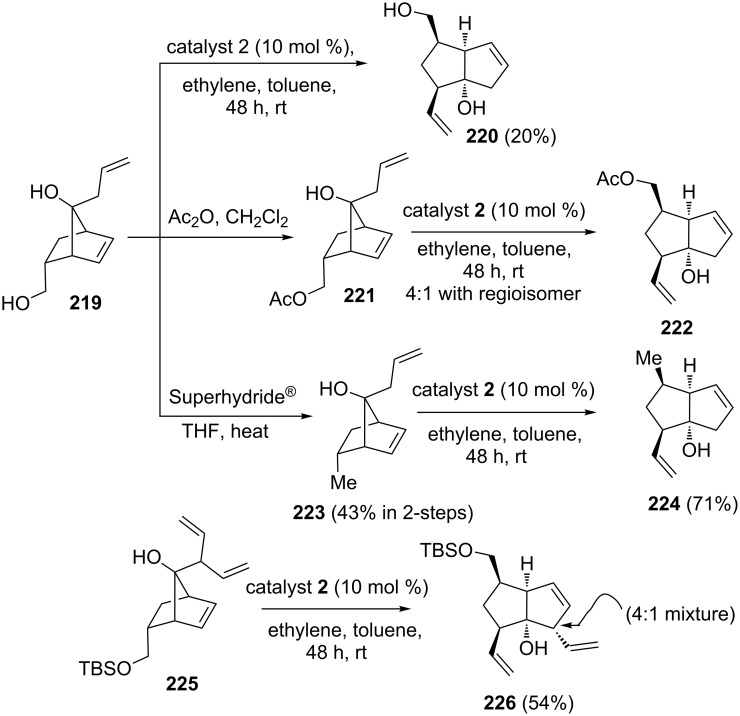
RRM strategy toward *cis-*fused bicyclo[3.3.0]carbocycles.

The bicyclo[3.3.0]octene system represent a core structural unit present in several natural products. Kimber and co-workers [48] have utilized a RRM approach to generate *cis-*fused bicyclo[3.3.0]octene derivatives. In this regard, various norbornenyl derivatives **219**, **221**, **223** and **225** were subjected to RRM by treatment with catalyst **2** in the presence of ethylene (**24**) to generate various bicyclo[3.3.0]octene derivatives such as **220**, **222**, **224**, and **226** with high regioselectivity. The thermodynamic stability of the product is anticipated to play an important role in the observed regioselectivity of these transformations (Scheme 45).

**Scheme 46:**
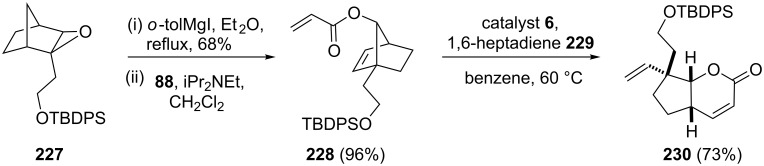
RRM protocol towards the synthesis of bicyclic lactone **230**.

In the course of the asymmetric synthesis of (−)-isoschizogamine, a bicyclic lactone **230** has been identified as a key building block. To this end, Fukuyama and co-workers [49] have used the RRM to generate the required building block **230**. In this reaction, the required norbornene derivative **228** was prepared from epoxide **227** in two steps and later it was treated with the more reactive catalyst **6** in the presence of 1,6-heptadiene (**229**) to generate the required bicyclic lactone **230** (73%). In this process, 1,6-heptadiene (**229**) helps to enhance the rate of the reaction and to improve the yield. However, when the bicyclic system **228** was treated with catalyst **5** in refluxing benzene lactone **230** was obtained in 24% yield (Scheme 46).

### Azanorbornene systems

**Scheme 47:**
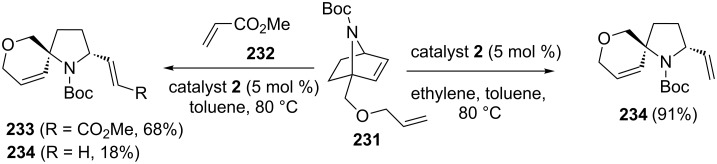
RRM approach to spiro heterocyclic compounds.

7-Azanorbornene derivatives have been used to generate a wide variety of heterocyclic compounds via the RRM approach [50]. To this end, the azanorbornene derivative **231** was treated with catalyst **2** in the presence of ethylene (**24**) to produce the heterospiro system **234** (91%). Alternatively, a ROM–RCM–CM sequence was employed under similar reaction conditions in the presence of methyl acrylate (**232**) as a CM partner. The tandem metathesis product **233** was obtained in 68% yield along with the ROM–RCM product **234** in 18% yield (Scheme 47).

**Scheme 48:**
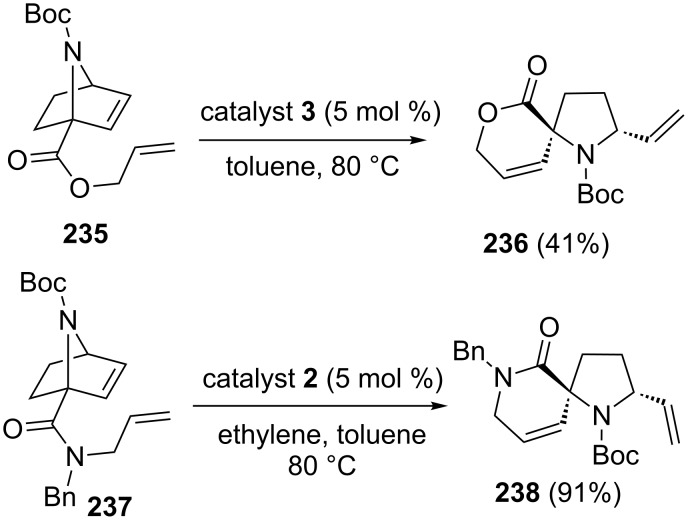
RRM approach to spiro heterocyclic compounds.

Later, 7-azanorbornene **235** has been used in RRM. To this end, compound **235** was subjected to a RRM under the influence of catalyst **3** to deliver the spiro heterocyclic compound **236** (41%). Similarly, compound **237** was treated with catalyst **2** under the same reaction conditions to produce the spiro heterocycle **238** (91%) (Scheme 48).

**Scheme 49:**
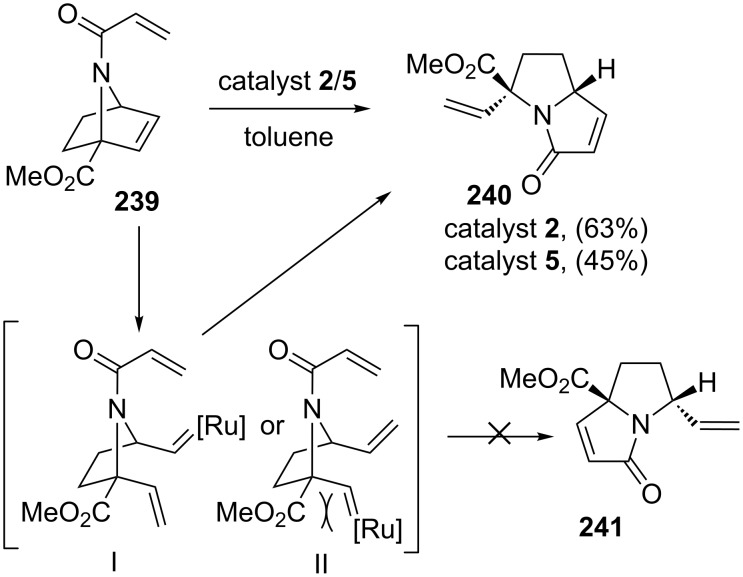
RRM approach to regioselective pyrrolizidine system **240**.

**Scheme 50:**
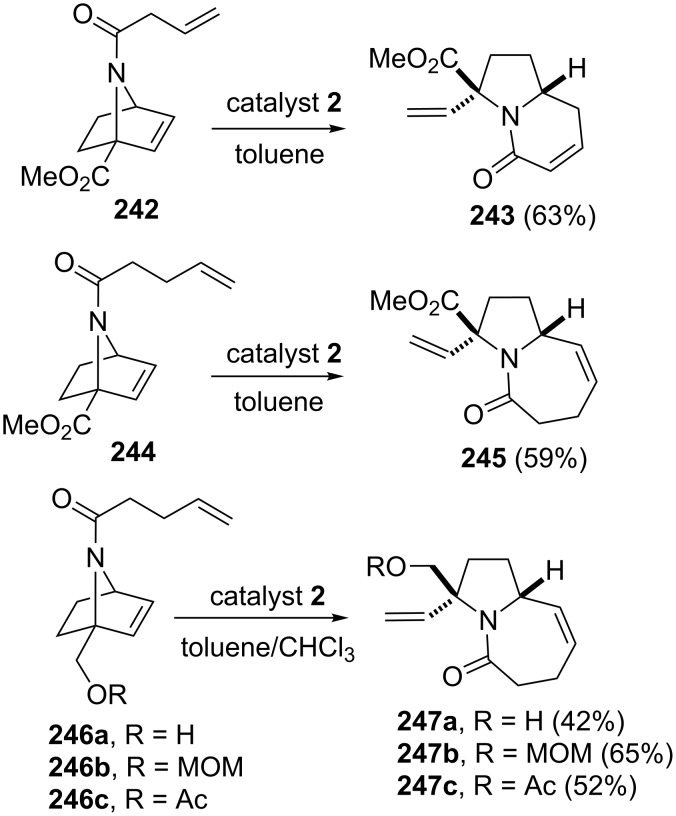
RRM approach to functionalized bicyclic derivatives.

The RRM strategy has provided an easy access to a variety of 1-azabicyclo[*n*.3.0]alkenones. For example, when 7-azanorbornene derivative **239** was subjected to a ROM–RCM sequence by treatment with catalyst **2** in toluene in the presence of ethylene (**24**) delivered the pyrrolizidine system **240** in 63% yield [51]. The regioselective formation of **240** may be attributed to the facile formation of a Ru–carbene intermediate where the metal participates on the side opposite to that of the methyl ester and thereby minimizing the steric crowding between ruthenium and carbonyl oxygen of an ester functionality (Scheme 49). Homologous starting material **242** underwent a RRM with catalyst **2** in the presence of ethylene (**24**) at 80 °C to produce indolizidine-based compound **243** in 63% yield. Under similar reaction conditions, the azabicyclic system **244** generated pyrrolo[1,2-*a*]azepine derivative **245**. When the RRM protocol was applied to compounds **246a–c** with different bridgehead substituents, they also generated the corresponding pyrrolo[1,2-*a*]azepine derivatives **247a–c** in good yields with a high degree of regioselectivity (Scheme 50).

**Scheme 51:**
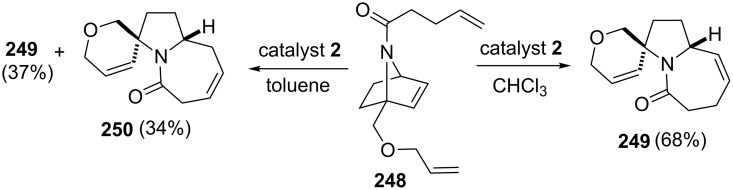
RRM approach to tricyclic derivatives **249** and **250**.

Treatment of ether-bridged triene **248** with catalyst **2** in chloroform at 50 °C generated the spiroannulated pyrrolidine **249** in 68% yield. However, when the reaction was performed in toluene at 80 °C, the isomeric tricyclic compound **250** was afforded in 34% yield and tricyclic derivative **249** was obtained in 37% yield (Scheme 51).

**Scheme 52:**
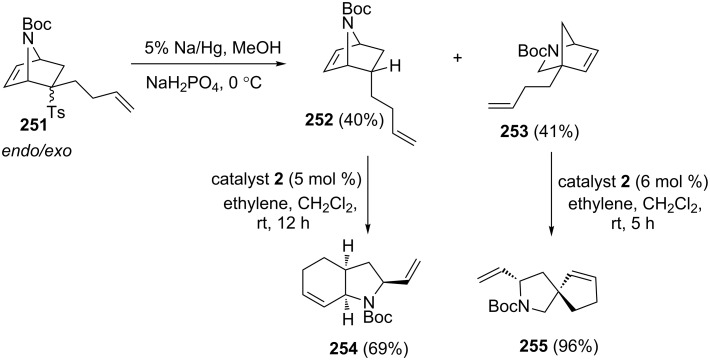
RRM approach to perhydroindoline derivative and spiro system.

Rainier’s group [52] has successfully demonstrated the synthesis of various perhydroindolines by adopting a ROM–RCM cascade using catalyst **2** starting with 7-azanorbornene derivative **251**. In this context, RRM precursors such as **252** and **253** were obtained from **251** by detosylation sequence. Later, they were subjected to a RRM protocol under the influence of catalyst **2** in the presence of ethylene (**24**) to generate the expected rearranged products **254** and **255**, respectively (Scheme 52 ).

### Oxanorbornene systems

**Scheme 53:**
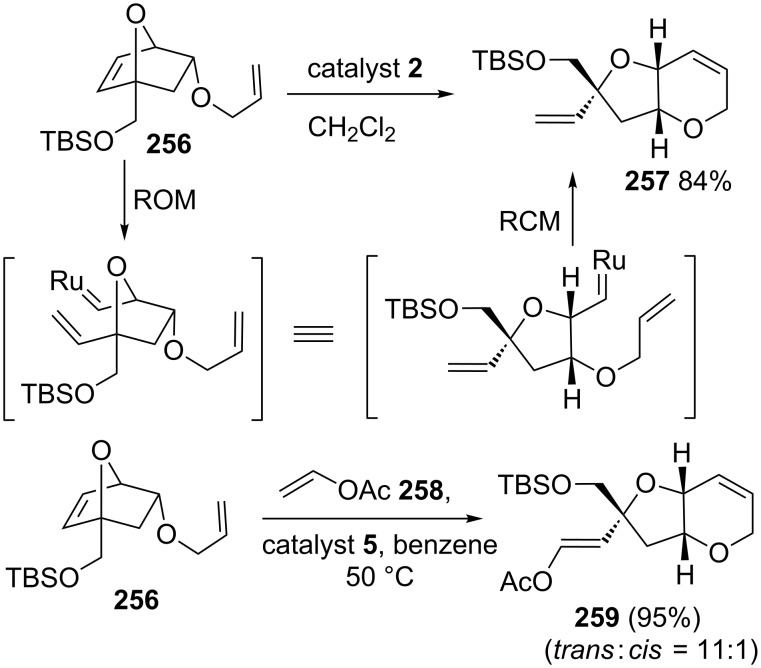
RRM approach to bicyclic pyran derivatives.

Lee and co-workers [53] have successfully constructed a fused bis(oxacyclic) system useful towards the formal total synthesis of dysiherbaine and neodysiherbaine via the RRM protocol. To this end, the oxabicyclo[2.2.1]hept-5-ene **256** was subjected to a RRM cascade with catalyst **2** in dichloromethane to produce pyran derivative **257** in 84% yield, which serves as a core structural unit of disyherbaine. Highly functionalized pyran derivative **259** was obtained by the reaction of **256** with catalyst **5** in the presence of vinyl acetate (**258**) (Scheme 53).

**Scheme 54:**
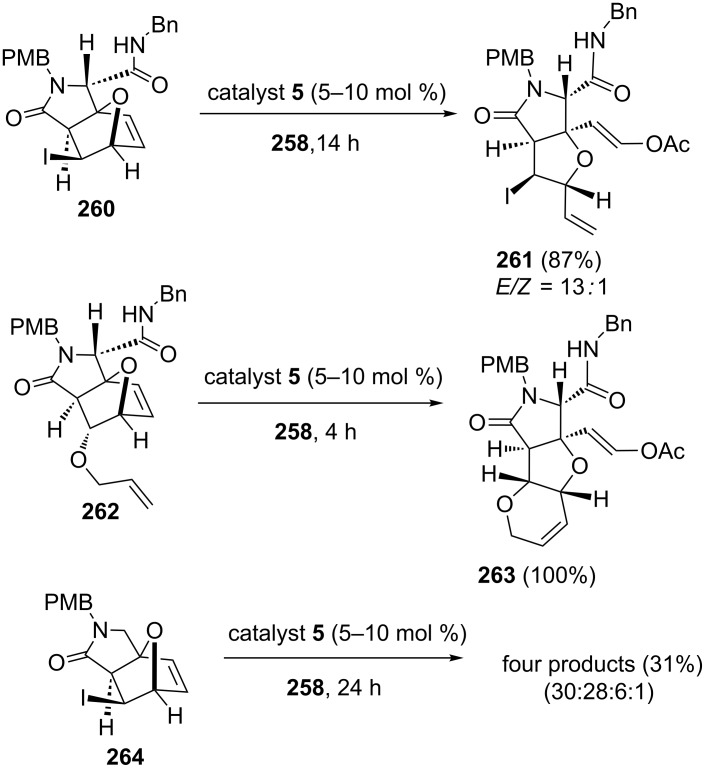
RRM of various functionalized oxanorbornene systems.

The RRM approach is useful to design diverse analogs of the marine toxin dysiherbaine, which displays antagonistic activity on ionotropic glutamate receptors from oxanorbornenes [54]. The report reveals the regiochemical directing effect of the exocyclic amidocarbonyl group in a ROM sequence of norbornenes. When the 7-oxanorbornene **260** containing an exocyclic amidocarbonyl moiety was subjected to a metathesis reaction using catalyst **5** in the presence of vinyl acetate (**258**) at room temperature, the required RRM product **261** was generated in 87% yield with high regio- (>99%) and good stereoselectivity (*E*/*Z* = 13:1). Next, tricyclic compound **263** was generated in quantitative yield when the oxanorbornene derivative **262** was subjected to a metathesis with catalyst **5** in the presence of vinyl acetate (**258**) at room temperature. On the other hand, when the norbornene derivative **264** without the *N*-benzylaminocarbonyl side chain was subjected to a metathesis under similar reaction conditions a mixture of four products (30:28:6:1) was obtained in 31% combined yield (Scheme 54).

**Scheme 55:**
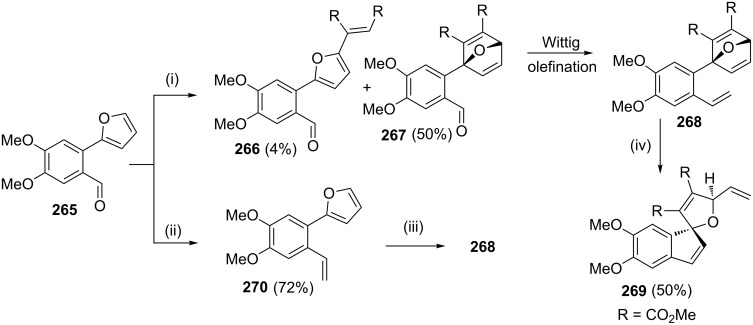
RRM to assemble the spiro fused-furanone core unit. (i) **129**, benzene, 55 °C, 3 days; (ii) Ph_3_P=CH_2_Br, *n*-BuLi, THF, 0 °C; (iii) **129**, benzene, 40 °C, 24 h; (iv) catalyst **2** (10 mol %), CH_2_Cl_2_, 35 °C.

Phelligridin G, a natural product isolated from the fruiting body of *P. igniarius,* is a well-known anticancer agent. To assemble the spiro-fused furanone core of phelligridin G, Wright and Cooper [55] have used a RRM process as a key step. Wittig olefination of furylbenzaldehyde derivative **265** using methyltriphenylphosphonium bromide in the presence of *n*-BuLi provided styrylfuran **270** in 72% yield. The DA reaction of styrene derivative **270** with DMAD **129** at 40 °C yielded oxabridged compound **268**. Another route to **268** involves a DA reaction of **265** with DMAD at 55 °C for longer reaction time (3 days) and sequential Wittig olefination. The spiro compound **269** was obtained from oxabicyclo adduct **268** by a domino metathesis sequence in the presence of catalyst **2**. Moreover, compound **269** was obtained as a single diastereomer and constitutes the core structure of phelligridin G (Scheme 55).

**Scheme 56:**
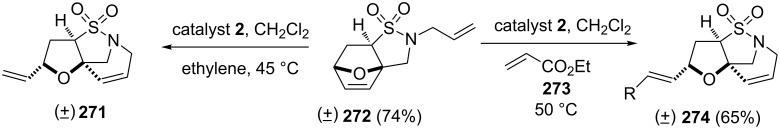
RRM protocol to norbornenyl sultam systems.

In 2009, Hanson’s group reported [56] the synthesis of skeletally diverse bi-, and tricyclic sultam derivatives (sulfonamide analogs) using norbornenyl sultam **272** as a core unit assembled by an intramolecular Diels–Alder (IMDA) reaction via a domino ROM–RCM–CM cascade. Diversity has been incorporated by using various cross-metathesis partners (Scheme 56).

**Scheme 57:**
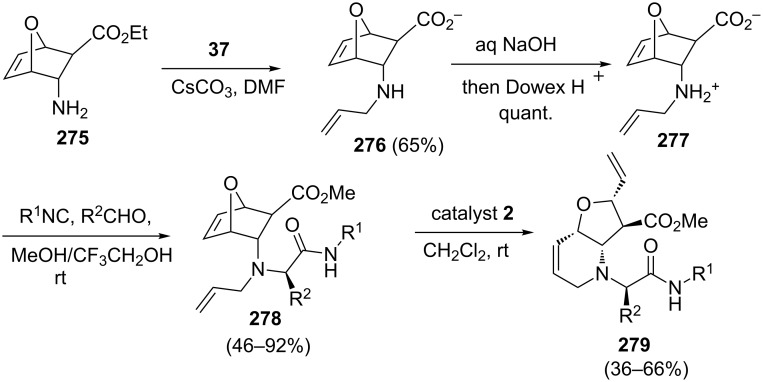
Ugi-RRM protocol for the synthesis of 2-aza-7-oxabicyclo system.

Basso and co-workers [57] have demonstrated a tandem Ugi–ROM–RCM protocol towards the synthesis of the 2-aza-7-oxabicyclo[4.3.0]nonane framework by employing catalyst **2**. They begin the synthesis with *N*-allyl-3-*endo*-amino-7-oxabicyclo[2.2.1]hept-5-ene-2-*exo*-carboxylic acid (**277**) and it was used in an Ugi 5-centre-4-component reaction (U-5C-4CR) with a wide variety of aldehydes and isocyanides. Subsequently, the products obtained (e.g., **2**7**8**) were subjected to a RRM protocol with catalyst **2** to generate the required 2-aza-7-oxabicyclo systems such as **279** (Scheme 57). The advantage of this approach is to provide a simple and short synthetic route to complex polycycles containing the 2-aza-7-oxabicyclo[4.3.0]nonane framework.

**Scheme 58:**
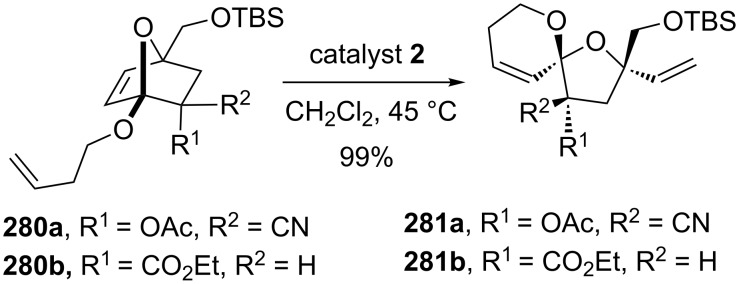
Synthesis of spiroketal systems via RRM protocol.

Blanchard and co-workers [58] have reported a novel protocol for the synthesis of spiro- and dispiroketals. The required oxabicyclic derivatives such as **280** were synthesized using α-alkoxyfurans by employing [4 + 2] and/or [4 + 3] cycloaddition reactions. Further, they used a RRM protocol in the presence of catalyst **2** to generate the spiroketal derivative **281** (Scheme 58).

**Scheme 59:**
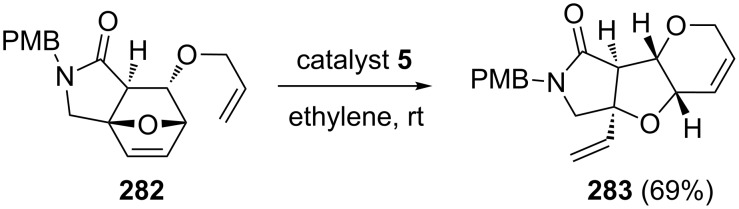
RRM approach to *cis*-fused heterotricyclic system.

Ikoma and co-workers [59] have reported a short synthetic sequence to *cis*-fused heterocycles by employing the 7-oxanorbornene system **282**. In this regard, compound **282** has been prepared by an intramolecular DA reaction as a key step and later, it was subjected to a RRM with catalyst **5** in the presence of ethylene (**24**) to generate the *cis*-fused heterotricyclic system **283** (Scheme 59).

**Scheme 60:**
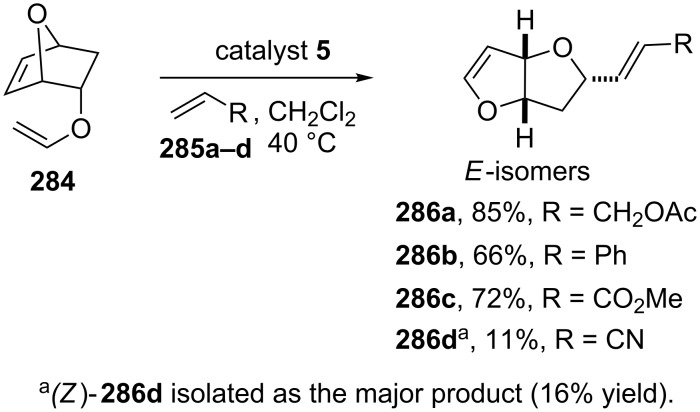
RRM protocol to functionalized bicyclic systems.

Quinn and co-workers [60] have demonstrated a simple approach to the synthesis of 2,6-dioxabicyclo[3.3.0]octenes **286** starting with the vinyl ether **284** derived from endo-7-oxanorbornene-2-ol by employing a tandem RRM–CM protocol (Scheme 60).

### Norbornene systems containing two heteroatoms

**Scheme 61:**
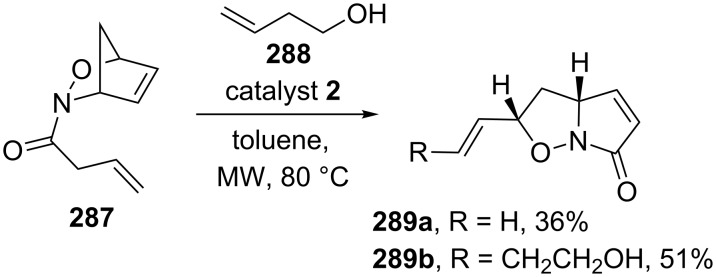
ROM/RCM/CM cascade to generate bicyclic scaffolds.

**Scheme 62:**
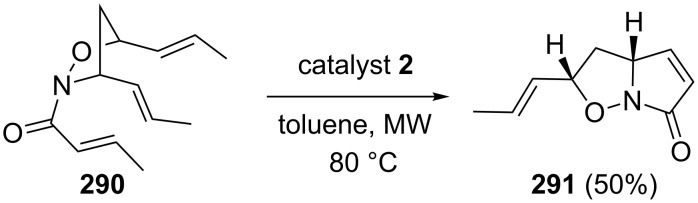
RCM of ROM/CM product.

Kouklovsky and Vincent have disclosed the RRM of nitroso Diels–Alder (NDA) adducts with a variety of alkenes under microwave or conventional heating conditions by employing catalyst **2** or catalyst **5** to generate various bicyclic compounds [61]. In this regard, compound **287** was subjected to a RRM cascade by employing catalyst **2** in the presence of but-3-en-1-ol (**288**) under optimized reaction conditions (MW, toluene, 80 °C) and the expected tandem metathesis product **289b** was obtained along with the ROM–RCM product **289a**. These compounds are useful synthones for the alkaloids synthesis (Scheme 61). In another instance, they also studied the efficiency of this method by isolating the RCM product of the ROM–CM byproduct **290**, which was recovered in the ROM–RCM–CM cascade (Scheme 62).

**Scheme 63:**
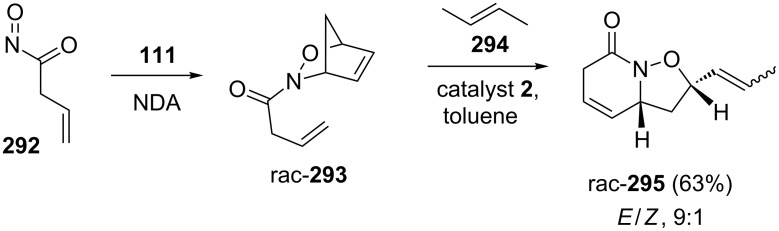
RRM protocol to bicyclic isoxazolidine ring system.

Kouklovsky and co-workers [62] have described a stereoselective synthesis of 2-(2-hydroxyalkyl)piperidine alkaloids by employing a RRM of NDA adduct **293**. The required building block **293** has been prepared via NDA reaction of compound **292** and cyclopentadiene (**111**). Later, the DA adduct was subjected to a RRM under the influence of catalyst **2** in the presence of but-2-ene (**294**) to generate the bicyclic isoxazolidine derivative **295**. By keeping the bicyclic isoxazolidine ring system intact, this protocol opened an efficient strategy for the formal synthesis of porantheridine and a total synthesis of andrachcinidine (Scheme 63).

**Scheme 64:**
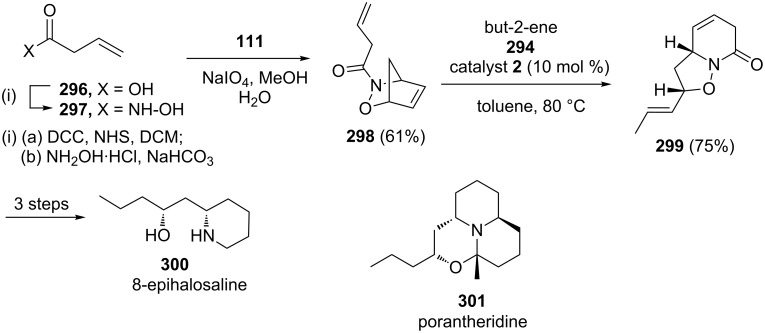
RRM approach toward the total synthesis of (±)-8-epihalosaline (**300)**.

They also reported the formal synthesis of (±)-porantheridine (**301**) and total synthesis of (±)-8-epihalosaline (**300**) via a sequential NDA reaction and a RRM [63]. The bicyclic compound **299** was identified as a key building block for the synthesis of 8-epihalosaline (**300**) and porantheridine (**301**). To this end, but-3-enoic acid (**296**) was converted to the required compound **297**, which on subjection to NDA in the presence of cyclopentadiene (**111**) furnished the desired cycloadduct **298** (61% overall yield). Later, it was subjected to the RRM cascade under the influence of catalyst **2** in the presence of **294** to obtain the desired precursor **299** (75% yield, Scheme 64).

### Bicyclo[2.2.2]octene systems

**Scheme 65:**
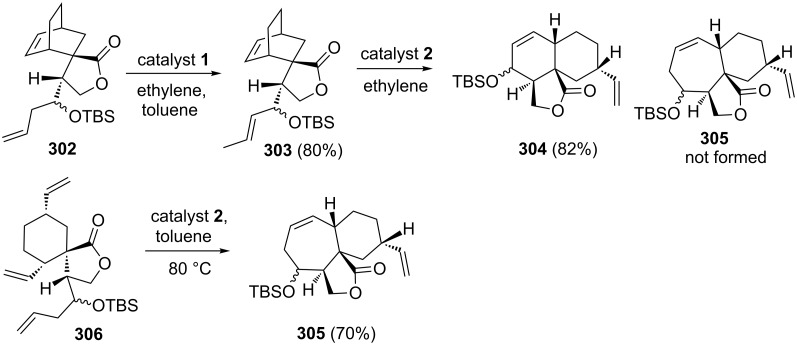
Sequential RRM approach to decalin **304** and 7/6 fused **305** systems.

Ghosh and co-workers [40] demonstrated that a RRM approach generates the decalin system **304** rather than the expected 7/6 fused bicyclic system **305**. The decalin system has been generated via ROM–RCM starting with bicyclo[2.2.2]octene derivative **303**. In this context, compound **302** was initially reacted with catalyst **1** in the presence of ethylene (**24**) to give **303**. Further, treatment with catalyst **2** gave the decalin derivative **304** rather than expected compound **305**. However, the metathesis of compound **306**, prepared by an independent route produced the expected RCM product **305** in 70% yield (Scheme 65).

**Scheme 66:**
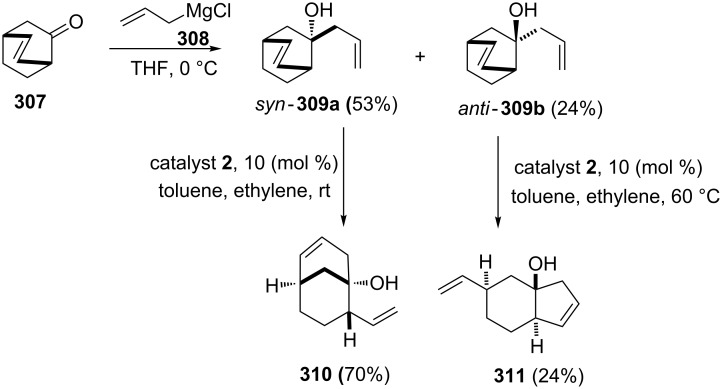
RRM protocol to various fused carbocyclic derivatives.

Kimber and co-workers [64] have described the synthesis of various carbocylic scaffolds by utilizing the RRM protocol involving catalyst **2**. They identified the bicyclo[2.2.2]oct-2-en-7-one (**307**) as the key building block, which was transformed into a mixture of alcohols such as *syn*-**309a** and *anti*-**309b** in 53% and 24% yield, respectively as a separable diastereomeric mixture (dr, 2:1 ratio). To this end, the *syn*-product **309a** effectively gave the RRM product **310** related to the bicyclo[3.3.1] system with catalyst **2** in the presence of ethylene (**24**). Alternatively, the *anti*-product **309b** gave the corresponding *trans*-fused [4.3.0]nonene derivative **311** in 24% yield (Scheme 66).

**Scheme 67:**
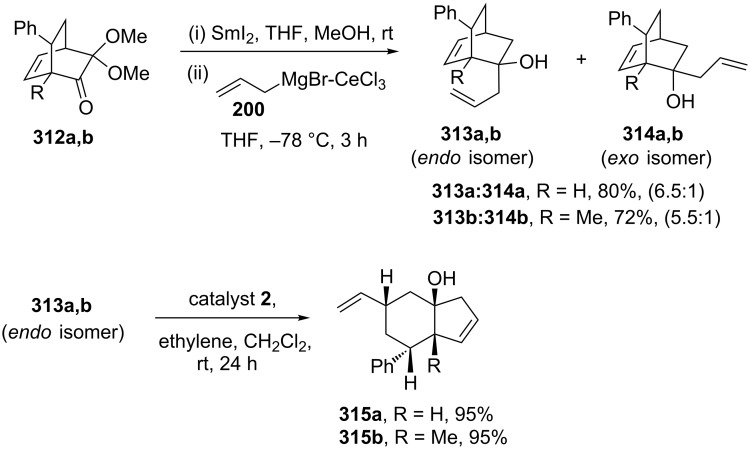
RRM to *cis*-hydrindenol derivatives.

Liao and co-workers [65] have employed the RRM protocol with the DA adduct derived from masked *o*-benzoquinones (MOBs). Here, they demonstrated an efficient RRM protocol for the synthesis of *cis*-hydrindenols starting with a readily available starting material such as 2-methoxyphenols. To this end, 2-allylbicyclo[2.2.2]octenol derivative **313** was identified as a key building block in the synthetic sequence, which was prepared from bicyclic system **312** in two steps. When the bicyclic compound **313** (*endo* isomer) was subjected to a RRM sequence with catalyst **2** in the presence of ethylene (**24**) at room temperature the desired *cis*-hydrindenols **315a** (95%), **315b** (95%) were obtained in excellent yield (Scheme 67).

**Scheme 68:**
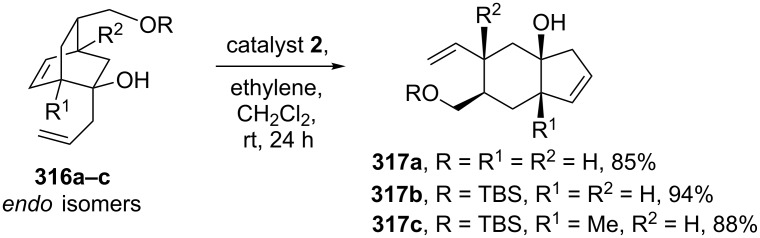
RRM protocol towards the *cis*-hydrindenol derivatives.

They have also shown that the RRM protocol is applicable with 2-allylbicyclo[2.2.2]octenol derivative **316**. The building block **316** required for this purpose has been generated via the DA reaction as a key step starting with 2-methoxyphenol. Later, compound **316** was subjected to a RRM under the influence of catalyst **2** in the presence of ethylene (**24**) to deliver the expected rearranged product **317** (Scheme 68).

**Scheme 69:**
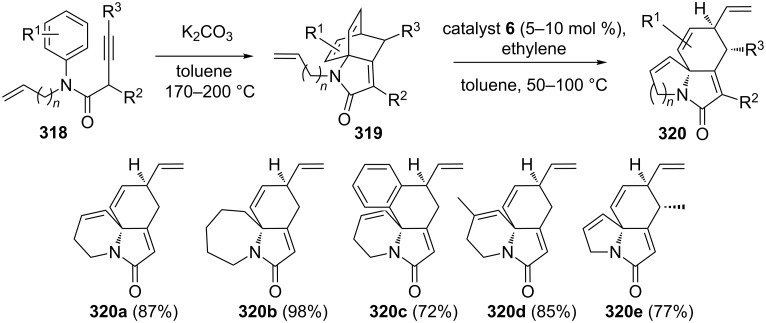
RRM approach toward the synthesis of diversed polycyclic lactams.

Vanderwal and co-workers [66] described the synthesis of polycyclic lactams obtained by arene/allene cycloaddition, discovered by Himbert and Henn were found to undergo a RRM in a facile manner in the presence of catalyst **6** to produce complex polycyclic lactams. In this regard, the required building block **319** was obtained from compound **318** by cycloaddition reaction. A variety of complex molecular frames were accessed via the RRM sequence under the influence of catalyst **6** in toluene at 50–100 °C in the presence of **24**. The procedure is suitable for the preparation of diverse polycyclic lactams with a variety of substitution patterns (Scheme 69).

**Scheme 70:**
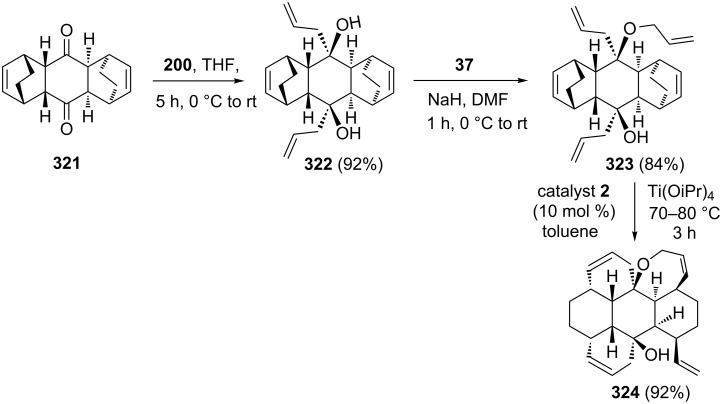
RRM approach towards synthesis of hexacyclic compound **324**.

Kotha and Ravikumar [44] have successfully executed the RRM protocol for the synthesis of condensed polycyclic systems. To this end, bicyclo[2.2.2]octene derivative **321** has been identified as a key starting material. The required key building block **323** has been prepared from the known bis-DA adduct **321** [67] via allyl Grignard addition followed by *O*-allylation sequence. The starting cycloadduct **321** was obtained by the double DA reaction between 1,3-cyclohexadiene and 1,4-benzoquinone. Further, treatment of **323** with catalyst **2** in the presence of titanium isopropoxide furnished the expected RRM product **324** in 92% yield (Scheme 70).

### Bicyclo[2.2.2]octene systems containing nitrogen

**Scheme 71:**
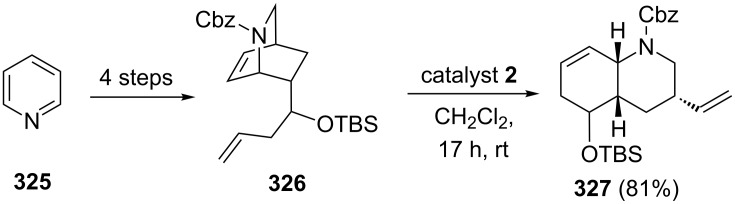
RRM protocol to generate luciduline precursor **327** with catalyst **2**.

To design lycopodium alkaloids, Barbe and co-workers [68] have used RRM judiciously. The required precursor **326** suitable for RRM has been prepared from pyridine (**325**) in four steps on gram scale. Later, the azabicyclic system was reacted with catalyst **2** to generate the desired hydroquinoline derivative **327** in 81% yield. Further, they have used the bicyclic compound **327** as a key building block in the total synthesis of (+)-luciduline (Scheme 71).

**Scheme 72:**
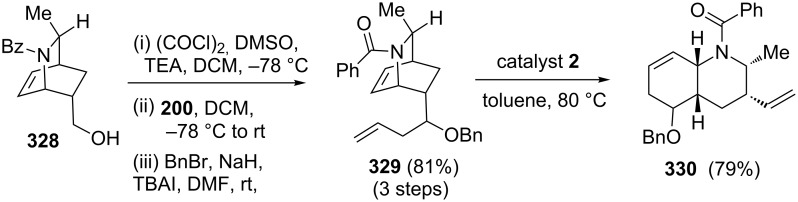
RRM protocol to key building block **330**.

Lepadins are natural products consisting of *cis*-fused decahydroquinoline subunits and they display cytotoxic activity against many human cancer cell lines. The total synthesis of (+)-lepadin B developed by Charette and Barbe [69] utilized a RCM–ROM as key step. In this regard the azabicyclic system **329** (obtained from pyridine (**325**)) was subjected to a RRM sequence by employing catalyst **2** at 80 °C in toluene to furnish the rearranged product **330** (79%). Further, the building block **330** was used in the stereoselective total synthesis of lepadin B (Scheme 72).

### Bicyco[3.2.1]octene derivatives

**Scheme 73:**
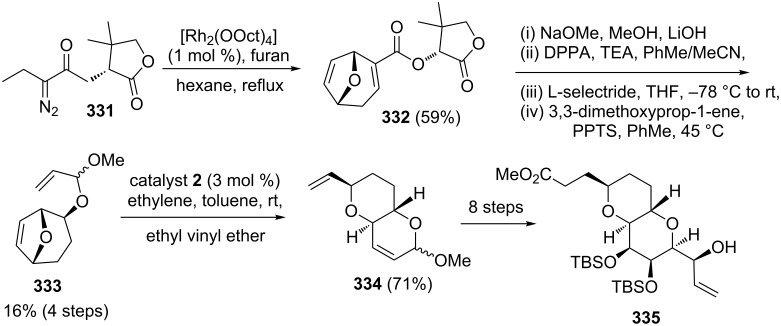
RRM approach towards the synthesis of key intermediate **335**.

Norhalichondrin B is a marine polyether belonging to the halichondrin family and its macrolactone analog has displayed anticancer activity. Phillips and co-workers [70] have described a total synthesis of norhalichondrin B in 37 steps from β-furylethanol. Interesting feature of this synthetic sequence is the tactical utilization of tandem ROM–RCM protocol towards the synthesis of the key intermediate **335**. In this reaction, the required RRM precursor **333** was obtained from diazo ester **331** in five steps. Further, the RRM of **333** with catalyst **2** furnished the required pyran derivative **334** (71%). Next, the fused ether **334** was transformed into the desired intermediate **335** in eight steps, which is a key intermediate required for the synthesis of norhalichondrin B (Scheme 73).

**Scheme 74:**
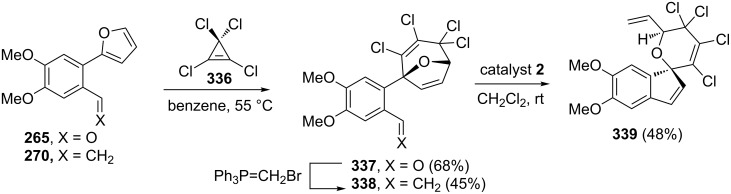
RRM protocol to highly functionalized spiro-pyran system **339**.

To expand the scope of the RRM methodology, Wright and Cooper [55] reported the synthesis of a highly functionalized pyran system by employing a RRM as a key step. To this end, 2-phenylfuran derivatives **265** and **270** were reacted with tetrachlorocyclopropene (TCCP, **336**) followed by olefination to result the required oxabicyclo[3.2.1]octene derivative **338**. Later, the RRM of the styrene derivative **338** with catalyst **2** delivered a highly-functionalized spiro-pyran derivative **339** in 48% yield (Scheme 74).

**Scheme 75:**
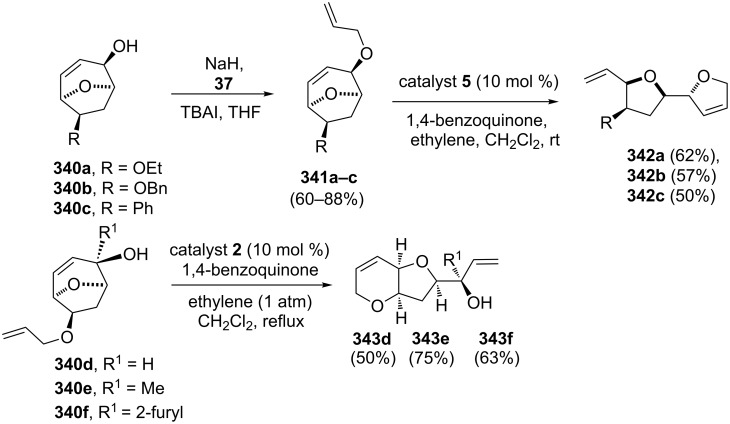
RRM to various bicyclic polyether derivatives.

The Dysiherbaine and acetogenin groups of natural products have been synthesized by the RRM approach. In this regard, secondary alcohol derivatives related to 8-oxabicyclo[3.2.1]octenes such as **341a,b**,**c** were used as potential precursors for the synthesis of a variety of cyclic polyethers [71]. Allylation of **340a–c** using sodium hydride and allyl bromide (**37**) in the presence of a phase-transfer catalyst such as tetrabutylammonium iodide generated bicyclic compounds **341a–c**. The RRM of these ether derivatives **341a–c** was performed under ethylene (**24**) atmosphere with catalyst **5** to generate the dihydrofuran derivatives **342a–c**. When compounds **340d**, **340e** and **340f** were subjected to a metathesis protocol by treatment with catalyst **2** under ethylene (**24**) atmosphere in the presence of 1,4-benzoquinone, *cis*-fused hexahydrofuro[3,2-*b*]pyran core containing compounds **343d**, **343e** and **343f** were obtained via RRM in good yields (50–75%) (Scheme 75).

## Conclusion

RRM involving ROM–RCM under the influence of various Ru–carbene complexes in one-pot sequence generate various complex targets. It is an atom economic process producing a wide range of polycyclic compounds containing highly demanding structures efficiently. Starting with relatively simple substrates, the final compounds obtained by the RRM process are generally difficult to synthesize by conventional synthetic routes. Various examples described here have clearly established the power and scope of this methodology. We believe that an increasing number of natural as well as non-natural products of high structural complexity have assembled by the RRM process and this activity will continue with more vigour in the future.
